# Laminin β4 is required for the development of human peripheral sensory neurons

**DOI:** 10.1242/dev.205371

**Published:** 2026-09-03

**Authors:** Kenyi Saito-Diaz, Tripti Saini, Archie Patel, Bhavik Dalal, Jayden Jackson, Christina James, Kimata Safi Thomas, Trinity Knight, Stephanie Beatrice Gogita, Kosuke Funato, Nadja Zeltner

**Affiliations:** ^1^ Center for Molecular Medicine, University of Georgia, Athens, GA 30602, USA; ^2^ Department of Biochemistry and Molecular Biology, University of Georgia, Athens, GA 30602, USA; ^3^ Department of Cellular Biology, University of Georgia, Athens, GA 30602, USA

**Keywords:** Human pluripotent stem cells, Peripheral nervous system, Sensory neurons, Laminin, Familial dysautonomia

## Abstract

The extracellular matrix (ECM) provides biophysical and biochemical cues necessary for cellular migration, differentiation and survival during development. Laminins are major ECM proteins consisting of α, β and γ chains. However, the function of laminin β4, encoded by *LAMB4*, remains unknown. Using human pluripotent stem cells (hPSCs), we characterize the role of *LAMB4* in human peripheral sensory neuron (SN) biology. We found that *LAMB4* is expressed during early SN specification, and it is required for SN development and survival. To assess clinical relevance, we examined familial dysautonomia (FD), a genetic disorder specifically affecting peripheral neurons. *LAMB4* variants previously identified in individuals with severe FD sharply downregulated *LAMB4* expression in SNs. Moreover, restoring a healthy ECM rescued the FD-related developmental phenotypes, suggesting that ECM defects contribute significantly to the etiology of FD. Finally, we showed that *LAMB4*/laminin β4 interacts with laminin α4 and laminin γ3 to form the previously unreported laminin-443 and is required for actin filament formation in SNs. Together, these results identify *LAMB4* as a crucial regulator of SN development and survival with clinical implications.

## Teaser

**Summary:** Human pluripotent stem cell studies reveal that *LAMB4* is required for sensory neuron development and that mutations in *LAMB4* may contribute to severe phenotypes in familial dysautonomia.

## INTRODUCTION

The extracellular matrix (ECM) is a dynamic network of proteins and glycoproteins that act as a cellular scaffold, providing the biophysical and biochemical cues necessary for cellular homeostasis (Bonnans et al., 2014; Frantz et al., 2010; Humphrey et al., 2014), migration, differentiation and survival during development (Rozario and DeSimone, 2010). This is of particular importance in neural crest cells (NCCs), which arise from the border of the neural plate and the non-neural ectoderm during development (Martik and Bronner, 2017; Simões-Costa and Bronner, 2015). NCCs migrate in a process regulated by ECM remodeling and morphogen gradients (Christiansen et al., 2000), giving rise to sensory neurons (SNs) and autonomic neurons (part of the peripheral nervous system), glial cells, endocrine cells and pigment cells, among others (Mayor and Theveneau, 2013). Changes in the biophysical properties of the ECM affect NCC differentiation (Zhu et al., 2019).

Laminins are major components of the ECM (Yap et al., 2019). They are heterotrimeric proteins consisting of α, β and γ chains expressed in different developmental stages for specific functions. For example, laminin-511 and laminin-111 are the most abundant laminins present during early development (Klaffky et al., 2001; Miner et al., 2004), whereas laminin-523 is expressed only in the retinal outer membrane (Pinzón-Duarte et al., 2010). Laminins interact with the transmembrane proteins integrins (Gardiner, 2011) to activate intracellular signaling pathways that reorganize the cytoskeleton (Domogatskaya et al., 2012).

There are five α chains, four β chains and three γ chains, which can potentially assemble up to 60 different trimers; however, only 16 have been identified (Domogatskaya et al., 2012). Of those, laminin β4 (expressed by the gene *LAMB4*) is understudied, and no laminin trimer containing the laminin β4 chain has been identified. Laminin β4 downregulation has been linked to diverticulitis, a disease of the peripheral nervous system (PNS) (Coble et al., 2017). Additionally, single-nucleotide variants of *LAMB4* have been identified in individuals with severe symptoms of the genetic disease familial dysautonomia (FD), which specifically affects peripheral neurons (Zeltner et al., 2016). Thus, we hypothesized that *LAMB4*/laminin β4 is necessary for the development and homeostasis of the PNS and its progenitors, the NCCs.

To address this, we use human pluripotent stem cell (hPSC) technology, which enables us to study cellular and molecular mechanisms of human development in cells from the endoderm, mesoderm and ectoderm (Joung et al., 2023; Vazin and Freed, 2010). Additionally, cells obtained from patients can be reprogrammed into induced pluripotent stem cells (iPSCs), which retain the same genetic background as the originating patients and are an invaluable tool for disease modeling (Zeltner and Studer, 2015).

Here, we have identified that *LAMB4* is expressed in the PNS and is necessary for NCC migration, and SN development and survival. Moreover, in the context of human *in vivo* development, we re-analyzed a comprehensive single-nucleus RNA-sequencing (snRNA-seq) atlas of the human fetal dorsal root ganglion (DRG), confirming that *LAMB4* expression is a genuine feature of human sensory neurogenesis *in vivo* and is absent in the rodent DRG. We also show that individuals with severe FD symptoms, who harbor mutations in *LAMB4*, exhibit significant laminin β4 downregulation. Additionally, we report that ECM deposited by healthy cells is sufficient to rescue the developmental phenotypes observed in FD. Finally, we show that laminin β4 forms the laminin-443 trimer, and it is required for the formation of actin filaments (F-actin) in SNs. Together, our results confirm that the ECM is necessary for SN development and survival, and that laminin β4 plays a crucial role in this process.

## RESULTS

### Laminin β4 is expressed in early stages of sensory neuron differentiation *in vitro*

**Fig. 1.**
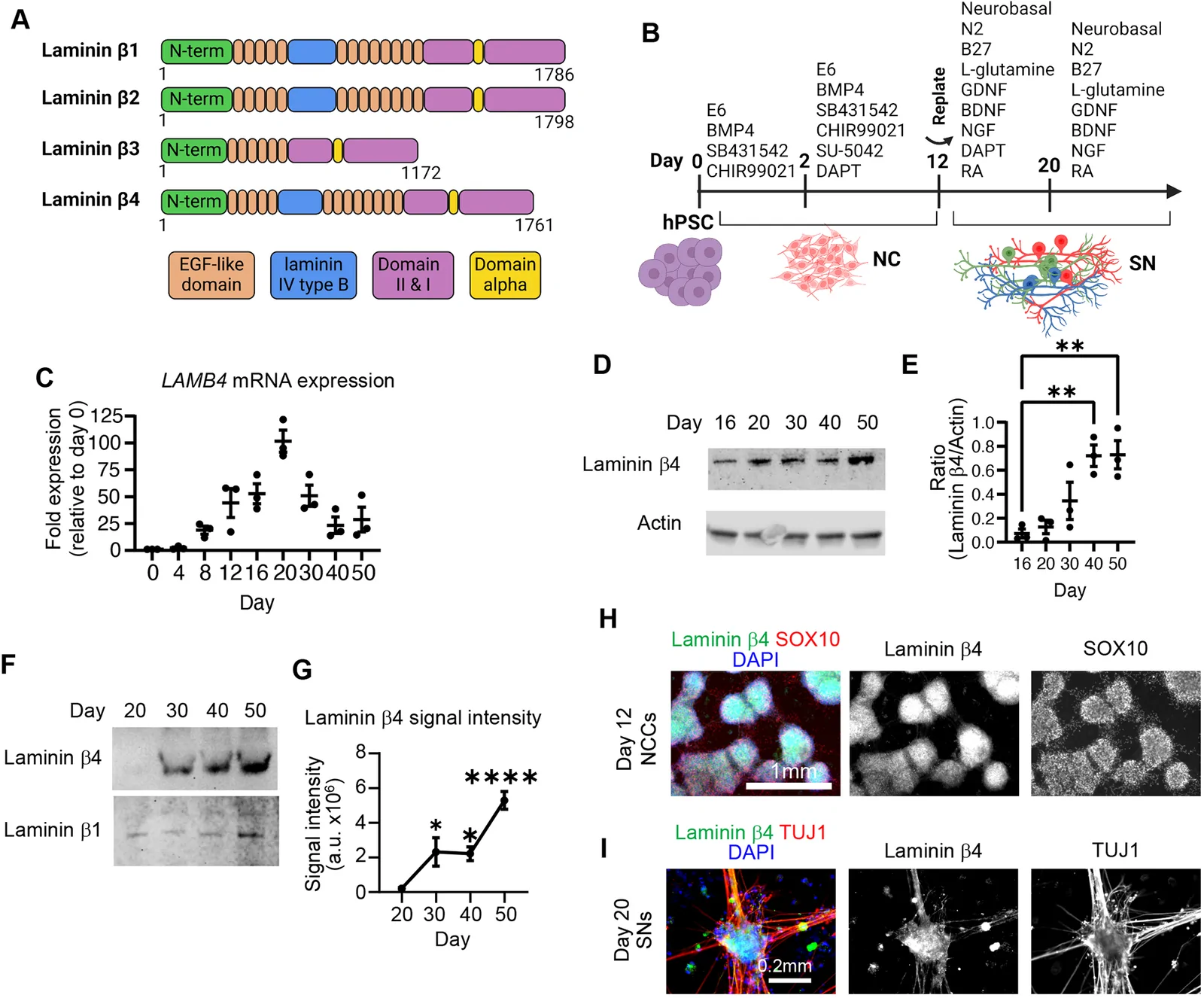
***LAMB4* is expressed in neural crest cells and sensory neurons.** (A) Comparison of human laminin β chains. (B) Schematics of the NC and SN differentiation protocol. (C) *LAMB4* mRNA expression from hPSC-ctr-H9 SNs measured by RT-qPCR (*n*=3 biological replicates). (D) Laminin β4 levels from cell lysates during SN development. Total protein was isolated from hPSC-ctr-H9 SNs and immunoblotted for laminin β4 and actin. (E) Signal intensity of immunoblots from D was quantified and normalized to day 16 (*n*=3 biological replicates). (F) Levels of laminin β4 in the ECM of SNs. ECM of hPSC-ctr-H9 SNs was collected and immunoblotted for laminin β4. Plates were coated with laminin β1 and were used as a loading control. (G) Signal intensity of immunoblots from F was quantified and normalized to day 20 (*n*=4 biological replicates). (H,I) Laminin β4 expression in NCCs and SNs. hPSC-ctr-H9 NCCs (day 12) and SNs (day 20) were fixed and stained for laminin β4 and DAPI. SNs were additionally stained for TUJ1. (C,E,G) One-way ANOVA followed by Dunnett's multiple comparisons test. ns, non-significant, *P<0.05, **P<0.005, ***P<0.001, ****P<0.0001. Data are mean±s.e.m. (A,B) Created in BioRender by Zeltner, N. (2006). https://BioRender.com/oo48aqb. This figure was sublicensed under CC-BY 4.0 terms.

To understand the biological function of laminin β4, we assessed the similarity between laminin β chains. We started by asking whether metazoans express laminin β4. To do this, we generated a phylogenetic tree comparing the amino acid sequences of laminin β chains expressed in multiple species (Fig. S1A). We found that rodents (*M*. *musculus* and *R*. *norvegicus*) do not have a *LAMB4* ortholog, in contrast to other vertebrates (*X*. *tropicalis*, *D*. r*erio*, *C*. *lupus familiaris* and *G*. *gallus*, Fig. S1A). Our analysis also showed that laminin β4 is closely related to laminin β1 and β2 (Fig. S1A). Moreover, only seven of the species analyzed have *LAMB4* orthologs, which could explain its limited *in vivo* characterization. We also found that laminin β4 shares structural similarities with laminin β1 and β2 within the N-terminal domain and EGF-like domains (Fig. 1A, Fig. S1B). These domains are important for laminin network assembly, suggesting that they could bind similar proteins. In contrast, laminin β3 showed the shortest amino acid sequence with only six EGF-like domains. On the C-terminal region, there were major differences in the amino acid sequences (Fig. 1A, Fig. S1C). The C-terminal region binds to the α and γ chains; thus, the differences between β chains might provide specificity during laminin assembly and be involved in the different affinities observed between laminin chains (Yao, 2017). We next sought to evaluate the lineage-specific expression pattern of *LAMB4*. We first differentiated control hPSC-ctr-H9 cells into definitive endoderm and mesoderm/cardiomyocytes, confirming robust expression of lineage-specific markers in each case, but not *LAMB4* (Fig. S2A-G). These findings were confirmed by reanalysis of published RNA-seq datasets from hPSC-derived endoderm (Loh et al., 2014) and mesoderm (Loh et al., 2016). Despite high expression of *LAMA1*, *LAMA5*, *LAMB1*, *LAMB2*, *LAMC1* and *LAMC2*, in both lineages (green), *LAMB4* was not expressed in endoderm (red rectangle, Fig. S2C) or mesoderm (Fig. S2G).

We next investigated *LAMB4* expression in ectoderm-derived NCCs by following a protocol to differentiate hPSCs into SNs using chemically defined conditions (Saito-Diaz and Zeltner, 2022; Saito-Diaz et al., 2021) (Fig. 1B). In this protocol, SNs are differentiated by going through all the developmental stages observed *in vivo* (Saito-Diaz et al., 2021). We found that *LAMB4* mRNA is expressed in day 12 NCCs differentiated from hPSC-ctr-H9 cells, and it peaked in the early stages of SN specification, by around day 20 (Fig. 1C). In contrast, laminin β4 isolated from cell lysates and the ECM gradually increased and peaked at later stages of SN development (day 40-50, Fig. 1D,E). It is possible that this increase is caused by laminin β4 still being assembled and secreted to the ECM, although not transcribed at high rates. To test this, we measured laminin β4 levels in the ECM alone. To do this, we lysed and removed the cells using ammonium hydroxide and resuspended the undisturbed ECM followed by immunoblotting (Hellewell et al., 2017). Consistent with our previous results, laminin β4 increased over time, suggesting either continuous secretion or gradual protein buildup (Fig. 1F,G). We confirmed our results by immunofluorescence (Fig. 1H,I, Fig. S2H). Together, our data show that *LAMB4* is expressed in NCCs and SNs.

### Laminin β4 is expressed in human fetal sensory neuron lineage *in vivo* but absent in the mouse embryonic dorsal root ganglion

**Fig. 2.**
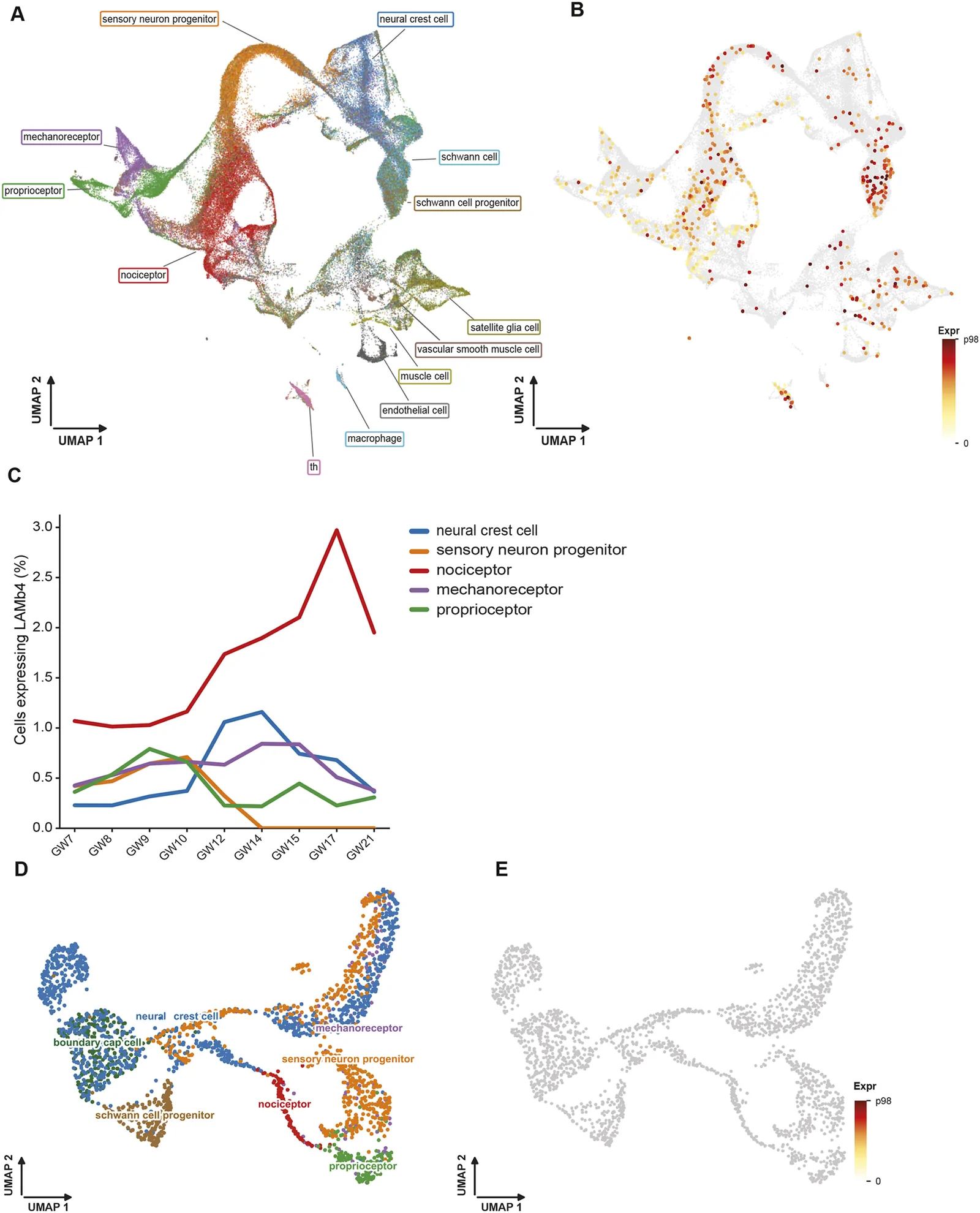
***LAMB4* is expressed in the human fetal SN lineage *in vivo* and is absent from the mouse embryonic DRG.** (A) Uniform manifold approximation and projection (UMAP) embedding of 98,323 cells from the human fetal DRG (GW7-GW21). (B) Global projection of *LAMB4* expression onto the human fetal DRG UMAP. *LAMB4*-positive cells (white to maroon) are significantly enriched within the SN lineage compared to non-sensory populations (Fisher's exact test, *P*=0.007). The maximum color threshold is set to the 98th percentile (p98) of expressing cells to mitigate outlier effects. (C) Spatiotemporal developmental trajectory of *LAMB4* penetrance across human gestation. Lines depict a rolling 2-point average of the percentage of cells expressing *LAMB4* (>0) for each major sensory cell type. Nociceptor penetrance rises steadily, peaking at ∼3% at GW17. (D) UMAP embedding of equivalent developing murine DRG (E9.5-E12.5). SN lineage and related progenitors are highlighted in color. (E) Global projection of *Lamb4* expression onto the murine DRG UMAP. *LAMB4* transcripts are absent (expression=0) across all identified cell populations, reflecting the genomic loss of the *LAMB4* ortholog in rodents.

To determine whether the spatiotemporal expression of *LAMB4* observed in our hPSC-derived SNs reflects bona fide human development, we analyzed a comprehensive snRNA-seq atlas of the human fetal DRG [gestational week (GW) 7-21] (Lu et al., 2024). *LAMB4* expression was confined almost exclusively to the SN lineage. Across 98,323 cells, *LAMB4*-positive cells were concentrated within neural crest cells (NCCs), SN progenitors (SNPs), nociceptors, mechanoreceptors and proprioceptors. Glial, vascular, stromal and immune populations remained largely negative (Fig. 2A,B). Consequently, *LAMB4* cells were significantly enriched in the sensory lineage compared to non-sensory cells (0.71% versus 0.57%; Fisher's exact test, *P*=0.007).

Temporal trajectory analysis demonstrated that *LAMB4* penetrance is highest in nociceptors, rising progressively from GW7 to a peak of ∼3% at GW17 before declining by GW21 (Fig. 2C). NCCs and SNPs maintained lower, sustained penetrance between GW8 and GW12, declining subsequently as the progenitor pool exhausts. The delayed surge of *LAMB4* in the nociceptor lineage during GW15-21 suggests a specialized role during nociceptor maturation.

To evaluate evolutionary conservation, we interrogated an equivalent embryonic mouse DRG dataset (E9.5-E12.5) (Faure et al., 2020). Despite robust representation of NCCs, SNPs and mature SN clusters, *Lamb4* transcripts were completely undetectable across all neural and glial populations (Fig. 2D,E). To confirm this was not a sequencing artifact, we verified robust spatial expression of the alternative laminin β isoforms (*Lamb1*, *Lamb2* and *Lamb3*) across the murine DRG (Fig. S2I-L). This transcriptional absence supports our phylogenetic analysis (Fig. S1A), confirming a genomic loss of the *Lamb4* ortholog in rodents. Together, these data establish that our hPSC-derived *LAMB4* expression program models a uniquely human feature of peripheral sensory neurogenesis rather than an *in vitro* artifact.

### Laminin β4 is required for neural crest cell migration and sensory neuron specification *in vitro*

**Fig. 3.**
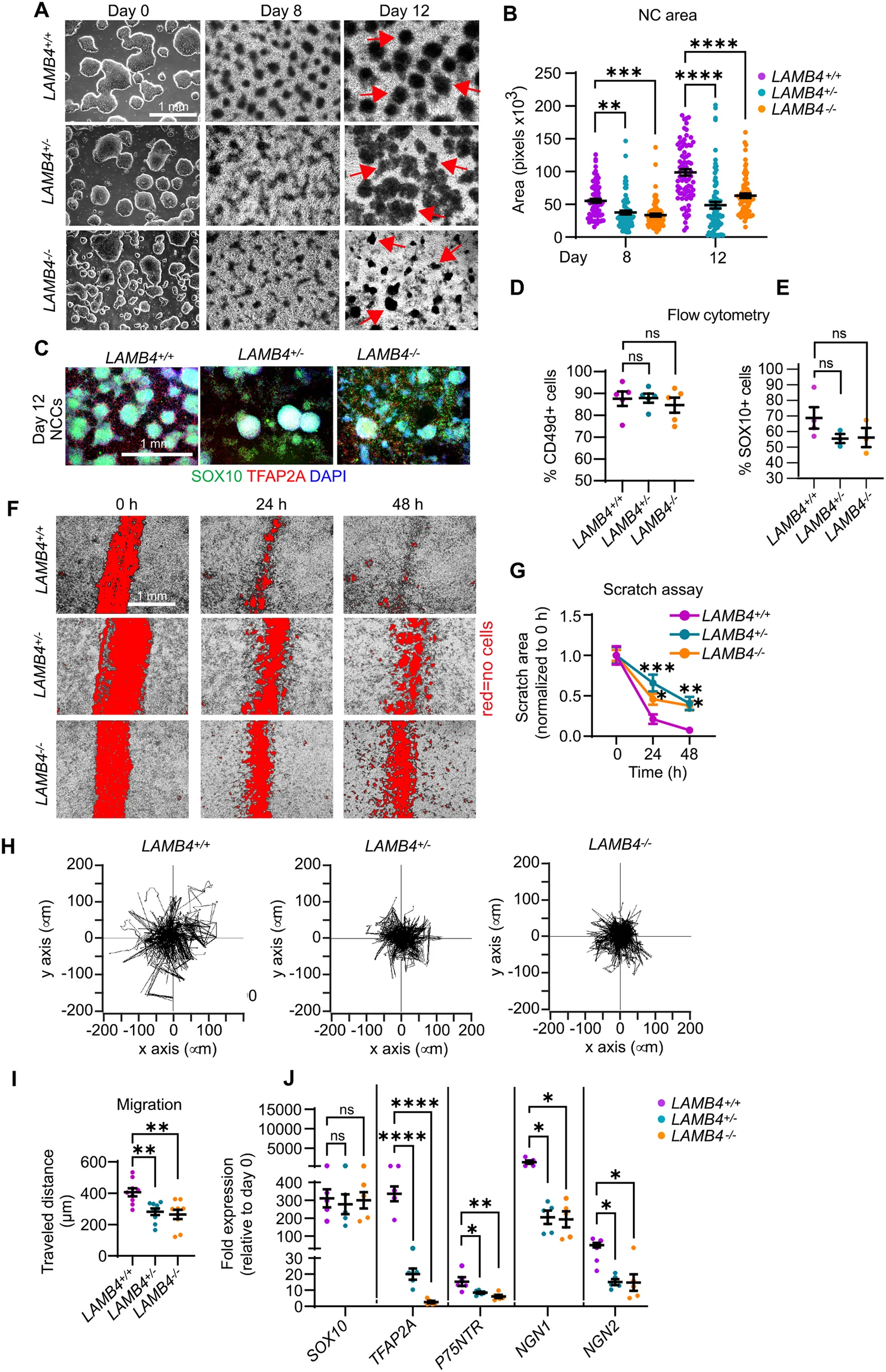
***LAMB4* is required for NCC migration.** (A) Bright-field images of *LAMB4^+/+^*, *LAMB4^+/−^* and *LAMB4^−/−^* hPSCs and NCCs colonies. Red arrows indicate NCC ‘clusters’. (B) Quantification of the area of clusters from A. Each dot indicates an individual cluster (>70 clusters, *n*=3 biological replicates). (C) NCC markers upon loss of *LAMB4*. *LAMB4^+/+^*, *LAMB4^+/−^* and *LAMB4^−/−^* NCCs were fixed and stained for SOX10 (green) and TFAP2A (red) and using DAPI (blue). (D,E) Number of NCCs differentiated from *LAMB4* mutant hPSCs. NCCs differentiated from *LAMB4^+/+^*, *LAMB4^+/−^* and *LAMB4^−/−^* cells were stained for (D) the NCC surface marker CD49d (*n*=5 biological replicates) and (E) the NCC intracellular marker SOX10 (*n*=3 biological replicates) quantified using flow cytometry. (F) NCC migration by scratch assay. NCCs from *LAMB4* mutants were replated on day 8 and scratched when they reached confluency. Bright-field images taken at 0, 24 and 48 h post-scratch (migrated area in red). (G) Scratched areas in F (red) were measured and normalized to 0 h. An average of 5-10 wells/condition are plotted (*n*=3 biological replicates). (H) NCC migration by live-cell imaging. Day 8 *LAMB4^+/+^*, *LAMB4^+/−^* and *LAMB4^−/−^* NCCs were replated and imaged every 10 min for 18 h. Individual cells were tracked, and their traveled distance and direction were measured and plotted. (I) Accumulated distance from H. The average of nine wells/condition are plotted (*n*=3 biological replicates). (J) NCC-related gene expression in *LAMB4^+/+^*, *LAMB4^+/−^* and *LAMB4^−/−^* NCCs (day 12) was measured using RT-qPCR (*n*=5 biological replicates). (B,D,E,I) One-way ANOVA followed by Tukey's multiple comparisons test. (J) One-way ANOVA followed by Dunnett's multiple comparisons test. (G) Two-way ANOVA followed by Tukey's multiple comparisons test. ns, non-significant, *P<0.05, **P<0.01, ***P<0.001, ****P<0.0001. Data are mean±s.e.m.

Next, we asked what is the role *LAMB4* plays in SN development. To address this question, we knocked out *LAMB4* in healthy hPSC-ctr-H9 cells using CRISPR/Cas9 (Fig. S3A). We identified a homozygous (*LAMB4^−/−^*) and a heterozygous (*LAMB4^+/−^*) clone (Fig. S3B). hPSC colonies showed no phenotypical differences compared to the parental cell line (*LAMB4^+/+^*, Fig. S3C); however, laminin β4 mRNA and protein levels were reduced in *LAMB4^−/−^* and *LAMB4^+/−^* SNs (Fig. S3D-F). Moreover, we did not find expression of closely related laminins α4, β1-4 and γ1 in *LAMB4^+/+^* and *LAMB4^−/−^* day 16 SNs, confirming that the CRISPR/Cas9-mediated knockout: (1) specifically targeted *LAMB4* (Fig. S3G) and (2) did not induce off-target effects or compensatory upregulation of related laminin family members. After confirmation, we used these cell lines to determine whether *LAMB4* is required for NCC development. We found that *LAMB4^−/−^* and *LAMB4^+/−^* can still differentiate into NCCs (Fig. 3A); however, the size of the characteristic ‘clusters’ formed by NCCs accumulation decreased (Saito-Diaz et al., 2021), as observed by bright-field microscopy (Fig. 3A, red arrows; Fig. 3B). To further confirm that these defects were caused by *LAMB4* loss, we reintroduced and/or overexpressed HA-tagged *LAMB4* in *LAMB4^−/−^* cells using a doxycycline-inducible lentiviral system (hereafter *LAMB4^−/− OE +dox^* or *LAMB4^−/− OE −dox^*). Successful *LAMB4* reintroduction was confirmed by immunoblot in *LAMB4^−/− OE +dox^* but not in *LAMB4^−/− OE −dox^* (Fig. S3H)*.* Doxycycline-induced *LAMB4* expression (*LAMB4^−/− OE +dox^*) rescued the reduced clustered area observed in *LAMB4^−/−^* NCCs, directly establishing that the morphological defects are attributable to *LAMB4* loss (Fig. S3I,J).

Next, we hypothesized that the reduced area was due to a decrease in the number of NCCs. We first tested this by immunofluorescence and found a reduced number of large SOX10^+^ clusters in *LAMB4^−/−^* and *LAMB4^+/−^* NCCs, although there was a large number of single SOX10^+^ cells (Fig. 3C). We confirmed this using flow cytometry to quantify the number of cells expressing the migratory NCC markers CD49d and SOX10 (Fattahi et al., 2016; Lai et al., 2021; Saito-Diaz et al., 2021) (Fig. 3D,E), suggesting that the number of NCCs was not affected by *LAMB4*. During development, NCCs migrate and accumulate, forming ganglia (Marmigère and Ernfors, 2007). Because the number of NCCs did not change upon *LAMB4* loss, but we observed a high number of individual SOX10^+^ cells by immunofluorescence (Fig. 3C), we hypothesized that loss of *LAMB4* impairs NCC migration. Indeed, in a scratch assay, *LAMB4^+/−^* and *LAMB4^−/−^* NCCs migrated significantly less after 48 h compared to *LAMB4^+/+^* (Fig. 3F,G). This was confirmed by live-cell imaging, where *LAMB4^+/−^* and *LAMB4^−/−^* NCCs showed reduced accumulated migration distance compared to the control (Fig. 3H,I). We next determined whether reduced migration in *LAMB4^+/−^* and *LAMB4^−/−^* NCCs also increased cell death. We performed cleaved caspase 3 immunostaining on NCCs from all three cell lines. Caspase 3 signal intensity was significantly increased in *LAMB4^+/−^* and *LAMB4^−/−^* cells compared to *LAMB4^+/+^*, suggesting that *LAMB4* loss also promotes apoptosis in NCCs, which might negatively impact the downstream SN differentiation (Fig. S3K,L). Finally, we characterized the expression of NCC genes in *LAMB4^+/−^* and *LAMB4^−/−^* cells. We found that *SOX10* expression was similar in all cell lines (Fig. 3J), consistent with our flow cytometry results (Fig. 3D,E). In contrast, *TFAP2A*, a NCC marker expressed prior to *SOX10*, was markedly reduced in *LAMB4^+/−^* and *LAMB4^−/−^* cells, suggesting that *LAMB4* might also affect the earliest stages of NCC specification. However, since SOX10^+^ and CD49d^+^ NCC numbers remained unchanged, sufficient cells were still committed to NCC fate. Interestingly, *tfap2a* downregulation has been shown to induce NCC apoptosis and impaired differentiation in zebrafish, a phenotype mirrored in our model (Barrallo-Gimeno et al., 2004). Additionally, SN specification genes downstream of *SOX10*, including *P75NTR* (*NGFR*), *NGN1* (*NEUROG1*) and *NGN2* (*NEUROG2*), were significantly downregulated (Fig. 3J), indicating that *LAMB4* plays a crucial role in directing NCCs toward an SN fate. Whether the downregulation of SN specification genes in *LAMB4-*deficient NCCs is a direct effect of *LAMB4* loss or is secondary to the migration defect remains to be determined. Together, our results show that *LAMB4* is necessary for NCC migration, which may in turn be required for proper SN differentiation and specification.

### Laminin β4 is required for the development of sensory neurons *in vitro*

**Fig. 4.**
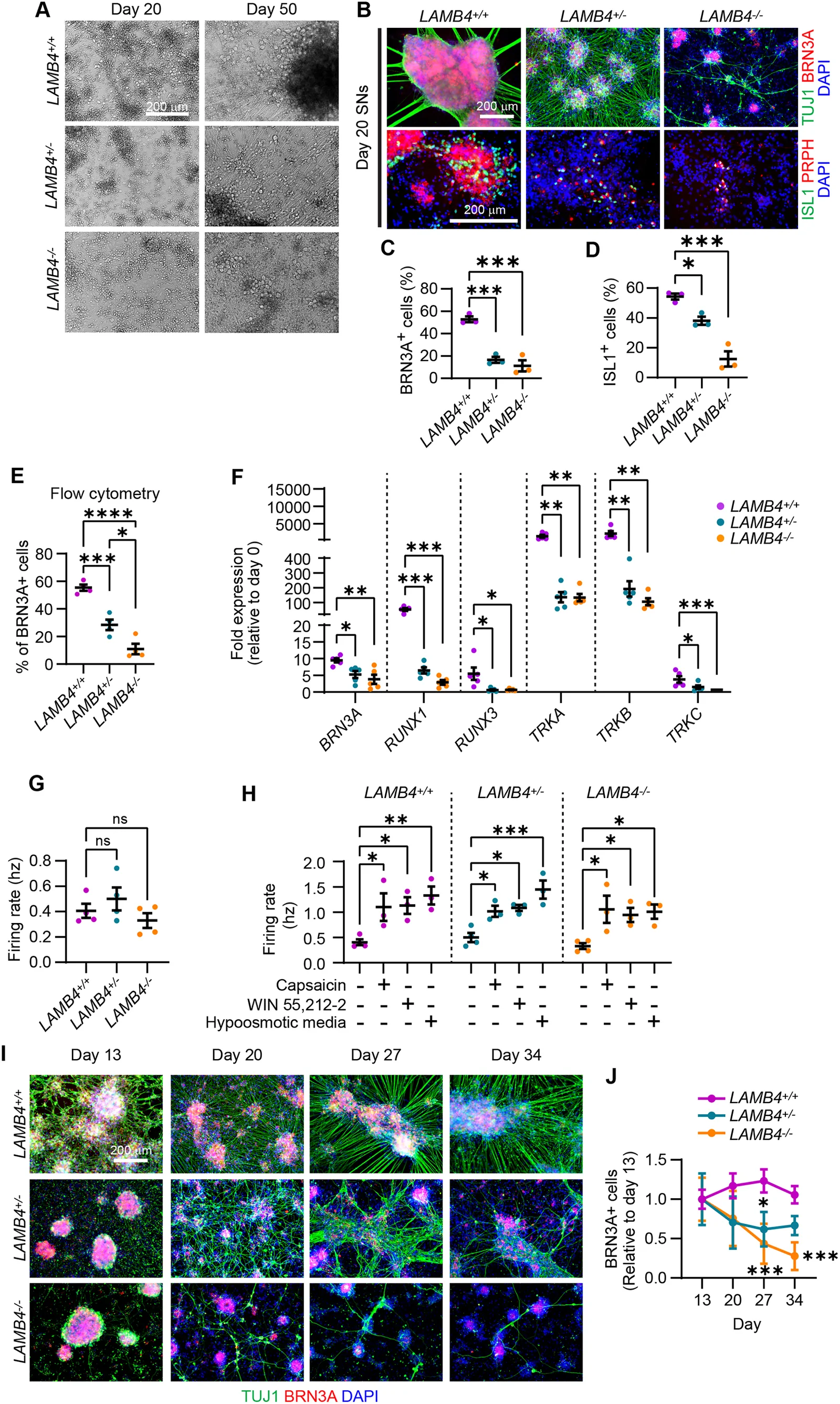
***LAMB4* is necessary for SN development and survival.** (A) Bright-field images of SNs differentiated from *LAMB4^+/+^*, *LAMB4^+/−^* and *LAMB4^−/−^* hPSCs. (B) Expression of SN markers upon *LAMB4* loss. Day 20 SNs were fixed and stained for TUJ1 (neuronal marker), PRPH (peripheral neuron marker), BRN3A and ISL1 (SN markers), and using DAPI (nuclei marker). Merged images are shown; individual channels are shown in Fig. S4A,B. (C,D) Percentage of (C) BRN3A^+^ and (D) ISL1^+^ cells from B. Data are normalized to DAPI. (E) Quantification of BRN3A^+^ SNs by flow cytometry from *LAMB4^+/+^*, *LAMB4^+/−^* and *LAMB4^−/−^* cells on day 20 (*n*=4 biological replicates). (F) SN expression markers in *LAMB4^+/+^*, *LAMB4^+/−^* and *LAMB4^−/−^* SNs on day 20 measured by RT-qPCR (*n*=5 biological replicates). (G) Electrical activity of *LAMB4* mutant SNs. Firing rate was measured using a multi-electrode array (MEA). Each dot represents the mean firing rate of six wells measured over 40 days (*n*=4 biological replicates). (H) Electrophysiological responses of SNs to activators. *LAMB4* mutant SNs were incubated with nociceptor agonists (0.25 μM capsaicin and 1 μM WIN55,212-2) and a mechanoreceptor activator (hypo-osmotic medium). Electrical activity was measured using MEA. Each dot represents the mean firing rate of six wells measured over 40 days (*n*=4 biological replicates). (I) Degeneration of *LAMB4^+/+^*, *LAMB4^+/−^* and *LAMB4^−/−^* SNs. Cells were cultured in plates coated with fibronectin and poly-L-ornithine, and reduced NGF concentration (1 ng/ml). Cells were fixed and stained for TUJ1 and BRN3A, and using DAPI. (J) Quantification of BRN3A^+^ SNs from I (*n*=3 or 4 biological replicates). (C-E,G) One-way ANOVA followed by Tukey's multiple comparisons test. (F,H) One-way ANOVA followed by Dunnett's multiple comparisons test. (J) Two-way ANOVA followed by Šídák's multiple comparisons test. ns, non-significant; *P<0.05, **P<0.01, ***P<0.001, ****P<0.0001. Data are mean±s.e.m.

Our results show that *LAMB4* is required to direct NCCs into the SN fate. Thus, we asked whether the loss of *LAMB4* negatively affects the SN development in our human *in vitro* system. We observed a reduced number of *LAMB4^+/−^* and *LAMB4^−/−^* SNs on day 20 and decreased size of SN clusters, which was reminiscent of the ganglia observed *in vivo* (Sleigh et al., 2016), by day 50 (Fig. 4A). This was confirmed by analysis of BRN3A and ISL1 (SN markers), PRPH (peripheral neuron marker) and TUJ1 (pan-neuronal marker) by immunofluorescence (Fig. 4B-D, Fig. S4A,B). Moreover, restoring *LAMB4* expression in *LAMB4^−/− OE +dox^* but not in *LAMB4^−/− OE −dox^* was sufficient to rescue these phenotypes (Fig. S4C-E), confirming that the neural deficits observed are *LAMB4* dependent.

We next quantified the number of SNs by flow cytometry. We found that *LAMB4^+/−^* hPSCs differentiate more efficiently than *LAMB4^−/−^* cells, suggesting that *LAMB4* expression level is important for SN development (Fig. 4E). We also found an increased number of non-neuronal cells expressing alpha-smooth muscle actin (αSMA) differentiated from *LAMB4^+/−^* and *LAMB4^−/−^* hPSCs compared to the parental control (Fig. S4F,G). Furthermore, *ACTA2*, the gene expressing αSMA, was upregulated in *LAMB4^+/−^* and *LAMB4^−/−^* cells (Fig. S4H), but not genes expressed by sympathetic neurons (*ASCL1*), motor neurons (*MNX1*), enteric neurons (*EDNRB*), and other CNS cells (*OLIG2*; Fig. S4H). Together, our results suggest that in the absence of *LAMB4*, NCCs take a non-neuronal cell fate, and SN differentiation is impaired, agreeing with our hypothesis that *LAMB4* is necessary for SN development.

The main SN subtypes found in the human DRG are nociceptors, mechanoreceptors and proprioceptors (Marmigère and Ernfors, 2007). Since *LAMB4* is necessary for SN development, we asked whether its loss impacts a particular subtype. During development, SN specification to a unique subtype is driven by specific genes. All SNs express *BRN3A*, whereas *RUNX1* and *TRKA* are expressed by nociceptors during development. In contrast, mechanoreceptors and proprioceptors express *TRKB* (*NTRK2*) and *TRKC* (*NTRK3*), respectively, and their progenitors express *RUNX3* (Marmigère and Ernfors, 2007). We found low *BRN3A* (*POU4F1*) mRNA levels in *LAMB4^+/−^* and *LAMB4^−/−^* SNs compared to *LAMB4^+/+^* cells (Fig. 4F). Additionally, the genes expressed by nociceptors [*RUNX1* and *TRKA* (*NTRK1*)], and mechanoreceptors and proprioceptors (*RUNX3*, *TRKB* and *TRKC*) were also downregulated (Fig. 4F). Furthermore, the number of SNs expressing TRKA, TRKB and TRKC were reduced in *LAMB4*-mutant SNs (Fig. S4I). This suggests that *LAMB4* is required for the development of the three main SN subtypes. We next tested the electrical activity of *LAMB4*-mutant SNs. *LAMB4^+/+^*, *LAMB4^+/−^* and *LAMB4^−/−^* SNs fired at the same rate (Fig. 4G). Furthermore, the number, duration, frequency and intervals of the bursts were similar among all lines (Fig. S4J-M). Additionally, activation of nociceptors with the agonists capsaicin and WIN55,212-2 (Saito-Diaz et al., 2021) similarly increased the firing rate of all SNs (Fig. 4H). This was also observed when mechanoreceptors were activated using hypoosmotic medium (Saito-Diaz et al., 2021) (Fig. 4H). Thus, loss of *LAMB4* reduces SN differentiation efficiency but does not impact their electrophysiological phenotypes, suggesting that *LAMB4* is required for SN specification but not for functional maturation. It is also possible that other laminin isoforms compensate for the loss of *LAMB4* in SNs.

The ECM is crucial for neuronal homeostasis (Melrose et al., 2021), thus we next asked whether *LAMB4* is necessary SN survival. Our differentiation protocol has been optimized to ensure the development and survival of wild-type SNs (Saito-Diaz et al., 2024). To assess degeneration, we modified the protocol to accelerate degeneration in vulnerable, diseased lines, while maintaining robust cell survival in healthy SNs. This protocol consists of: (1) reducing the concentration of nerve growth factor (NGF) in the differentiation medium; and (2) not using laminin-coated plates during the differentiation (Saito-Diaz et al., 2024), which serves as an external stressor. With this approach, we found that *LAMB4* loss increased the rate of SN degeneration (Fig. 4I,J). Together, our results show that *LAMB4* is required for both development and survival, but not the function of SNs. Having established the fundamental role of *LAMB4* in SN development, we next asked whether altered *LAMB4* expression has clinical implications.

### A healthy extracellular matrix rescues the developmental phenotypes in severe FD *in vitro*

**Fig. 5.**
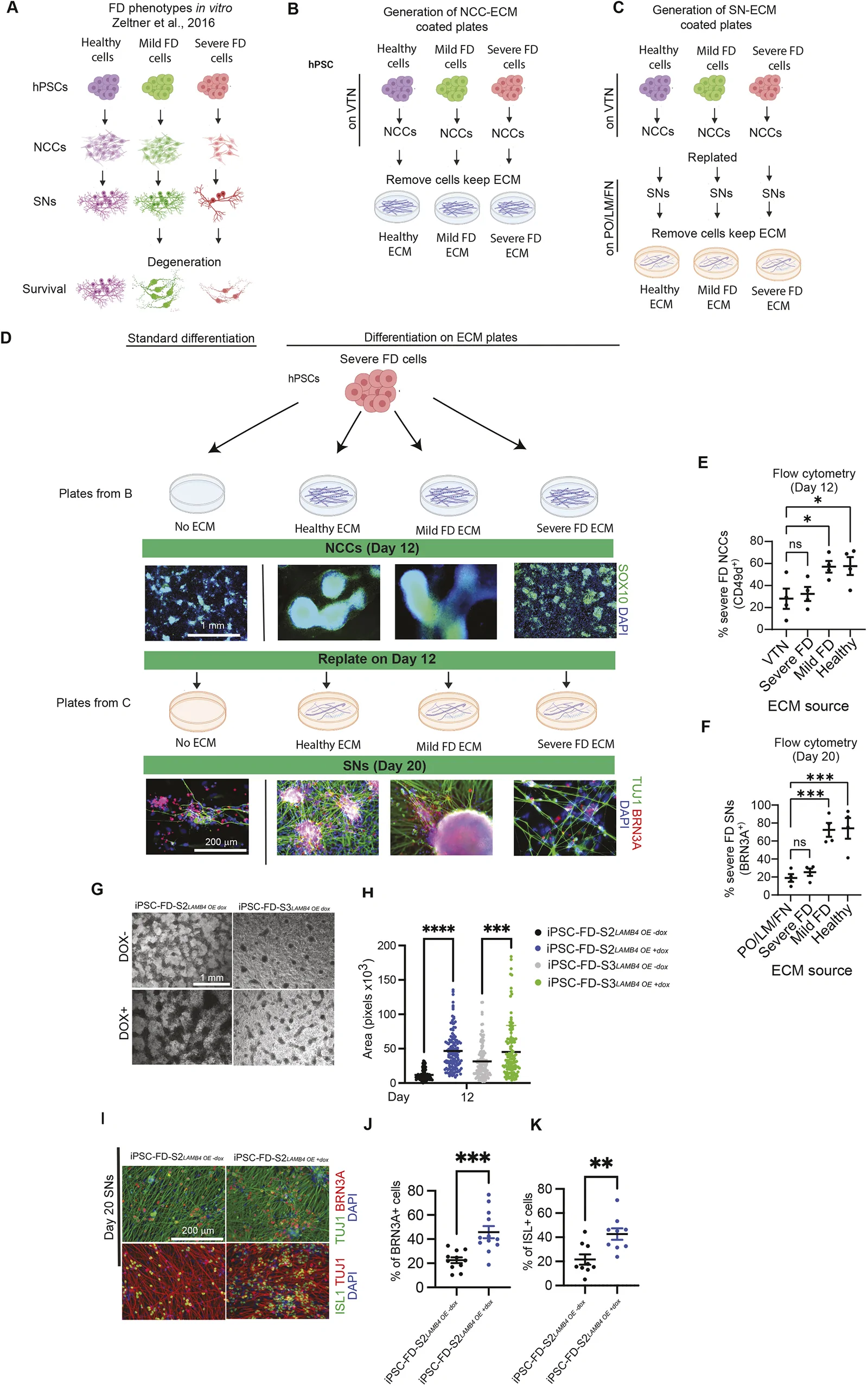
**Healthy ECM rescues the developmental defects from severe familial dysautonomia *in vitro*.** (A) Phenotypes of SNs differentiated from iPSCs of familial dysautonomia (FD) patients with mild versus severe symptoms. (B,C) Schematics of ECM rescue experiments at the NCC stage and at the SN stage. (D) Effects of the ECM in NCC and SN differentiation. iPSC-FD-S3 iPSCs were differentiated on vitronectin (VTN, standard differentiation) alone (left) or on ECM deposited by healthy, mild or severe FD NCCs (right). NCCs were fixed on day 12 and stained for SOX10 and using DAPI (top row). iPSC-FD-S3 NCCs were differentiated in dishes coated with poly-L-ornithine (PO), laminin-111 (LM) and fibronectin (FN) (standard differentiation) (left) or on ECM from healthy, mild or severe FD SNs (right). Day 20 SNs were fixed and stained for the neuronal marker TUJ1 and SN marker BRN3A (bottom row). (E) Quantification of NCCs from D. NCCs were stained for the NCC marker CD49d and analyzed using flow cytometry (*n*=4 biological replicates). (F) Quantification of SNs from D. SNs were fixed on day 20, stained for BRN3A and quantified using flow cytometry (*n*=4 biological replicates). (G) *LAMB4* overexpression rescues the severe FD phenotype in NCCs. Bright-field images of iPSC-FD-S2*^LAMB4 OE dox^* and iPSC-FD-S3*^LAMB4 OE dox^* colonies in the absence and presence of 1 μg/ml doxycycline at NCCs stage (day12) showing the NCCs clusters. (H) Area quantification of NCC clusters in G. (I) *LAMB4* overexpression rescues the severe FD phenotype in SNs. Expression of SN markers upon 1 μg/ml doxycycline treatment iPSC-FD-S2*^LAMB4 OE dox^.* SNs were fixed on day 20 and stained for TUJ1 and SN markers (BRN3A and ISL1). Nuclei were stained with DAPI. (J,K) Quantification of the percentage of (J) BRN3A^+^ and (K) ISL1^+^ cells in H. Data are normalized to DAPI. (E,F) One-way ANOVA followed by Dunnett's multiple comparisons test. (H) One-way ANOVA followed by Tukey's multiple comparisons test. (J,K) Unpaired two-tailed *t*-test with Welch's correction. ns, non-significant; *P<0.05, **P<0.01, ***P<0.001, ****P<0.0001. Data are mean±s.e.m.

FD is a genetic disease that specifically targets peripheral neurons (González-Duarte et al., 2023) and was one of the first diseases modeled using iPSC technology (Lee et al., 2009). It is caused by a mutation in the elongator complex scaffold protein *ELP1* (Cuajungco et al., 2003; Slaugenhaupt et al., 2004). Although 99.5% of individuals with FD share the same *ELP1* mutation, symptom severity varies widely. We have previously shown that symptom severity can be recapitulated in our *in vitro* system (Zeltner et al., 2016). SNs from mild-FD iPSCs showed only degeneration, while severe-FD iPSCs showed both developmental and degenerative defects (Zeltner et al., 2016) (Fig. 5A). Severely, but not mildly, affected FD patients also harbored *LAMB4* variants, accounting for the phenotypical difference (Table S1). Thus, we asked whether ECM composition drives this phenotype and whether a healthy ECM can rescue it. To test this, we performed an ECM-rescue experiment: healthy hPSC-ctr-H9, mild-FD (iPSC-FD-M2) or severe-FD (iPSC-FD-S3) cells were differentiated to the NCC stage. Cells were then removed, leaving behind their deposited ECM (Hellewell et al., 2017) (Fig. 5B). We also differentiated the same lines to SNs, removed the cells and kept the deposited ECM dishes (Fig. 5C). We then seeded severe-FD iPSCs (FD-S3) onto these ECM-coated plates (Fig. 5B,C) to test if the phenotype could be rescued (Fig. 5D). On standard plates, severe-FD cells generate few NCCs and SNs (Fig. 5D, left). As expected, healthy and mild-FD ECM rescued the NCC phenotype severe-FD iPSCs but not severe-FD ECM, which lacks functional *LAMB4* (Fig. 5D, right). This was confirmed by flow cytometry analysis for CD49D^+^ for NCCs and BRN3A^+^ for SNs (Fig. 5E,F), showing that a healthy ECM is crucial for NCC and SN development.

To confirm these results, we introduced doxycycline-inducible HA-tagged *LAMB4* into severe-FD lines: iPSCs-FD-S2 and iPSCs-FD-S3 (Fig. S5A, hereafter iPSC-FD-S2*^LAMB4 OE−dox^* or iPSC-FD-S2*^LAMB4 OE+dox^*, and iPSC-FD-S3 *^LAMB4 OE-dox^* or iPSC-FD-S3*^LAMB4 OE+dox^*, respectively). Interestingly, both iPSC-FD-S2*^LAMB4 OE+dox^* and FD-S3*^LAMB4 OE+dox^* showed significant increase in NCC clustering compared to uninduced iPSC-FD-S2*^LAMB4 OE-dox^* and iPSC-FD-S3*^LAMB4 OE-dox^* controls (Fig. 5G,H), showing that *LAMB4* is sufficient to rescue the developmental defect. At the SN stage, iPSC-FD-S2*^LAMB4 OE+dox^* also showed significantly more BRN3A^+^ and ISL1^+^ neurons than iPSC-FD-S2*^LAMB4 OE-dox^* controls (Fig. 5I-K), showing that *LAMB4* overexpression rescues this neural FD phenotype. Together, these results show *LAMB4* is a crucial ECM component required for SN development in severe FD.

### Laminin β4 is downregulated in sensory neurons affected by severe FD symptoms

**Fig. 6.**
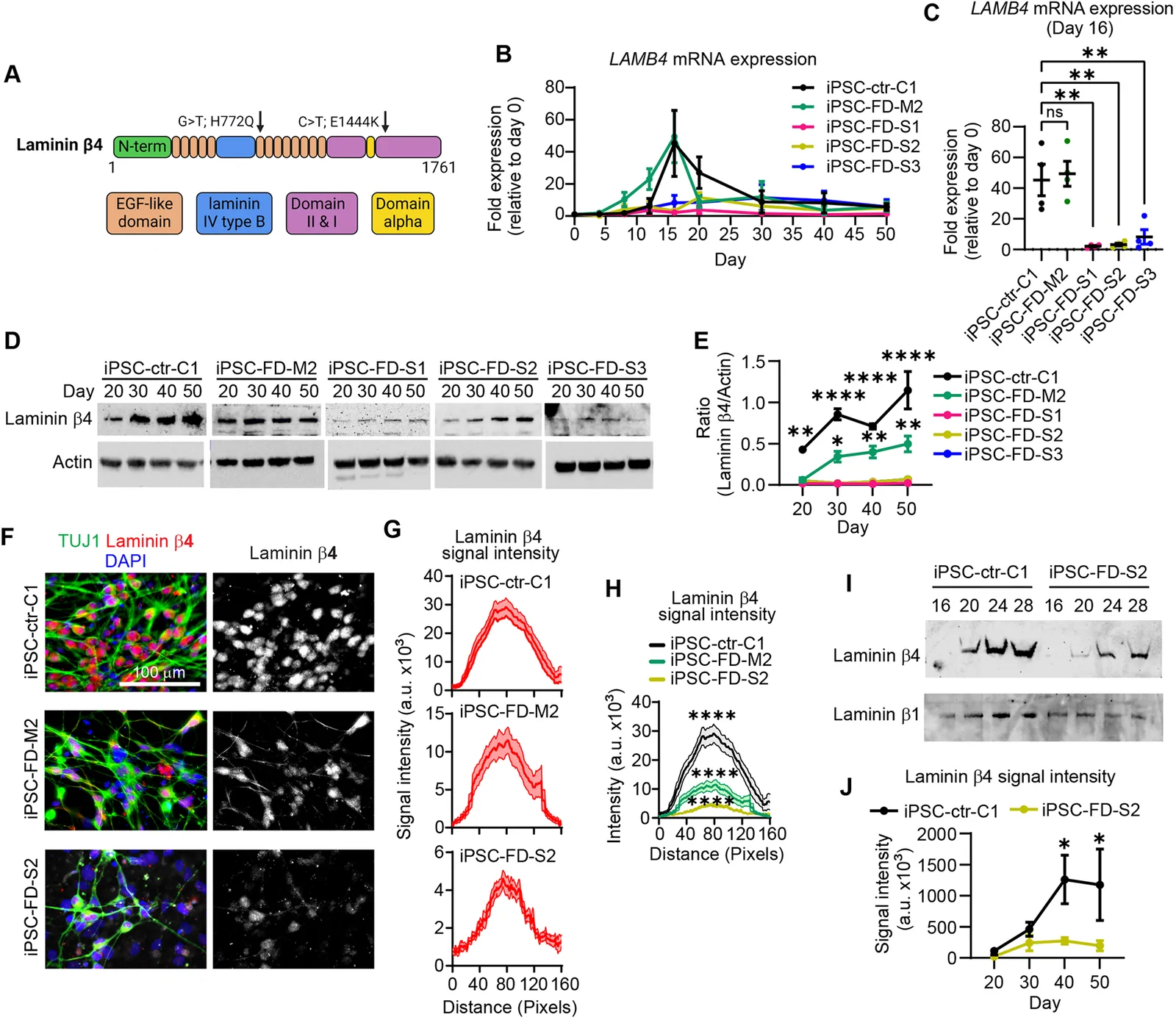
***LAMB4* expression is downregulated in individuals with severe familial dysautonomia symptoms.** (A) Schematic of the single nucleotide variants identified in *LAMB4* in individuals with severe familial dysautonomia (FD). (B) *LAMB4* expression in SNs differentiated from iPSCs of individuals with severe FD. Severe FD iPSC lines S1, S2 and S3, one mild FD iPSC line (M2), and one healthy control iPSC line (C1) were differentiated into SNs. Gene expression was measured by RT-qPCR (*n*=4 biological replicates). (C) *LAMB4* expression by day 16 SNs shown in B is shown (*n*=4 biological replicates). (D) Laminin β4 expression during SN development. Lysates from iPSC-FD-S1, iPSC-FD-S2, iPSC-FD-S3, iPSC-FD-M2 and iPSC-ctr-C1 SNs immunoblotted for laminin β4 and actin. (E) Quantification of signal intensity of immunoblots shown in D (*n*=3 biological replicates). Differences between iPSC-FD-S1, iPSC-FD-S2 and iPSC-FD-S3 versus iPSC-ctr-C1 or iPSC-FD-M2 were analyzed. (F) Laminin β4 in SNs. SNs from iPSC-FD-S2, iPSC-FD-M2 and iPSC-ctr-C1 cells were fixed on day 20 and stained for laminin β4 and TUJ1, and using DAPI. (G) Laminin β4 signal intensity measured from images in F. The average of 20 cells is plotted (*n*=3 biological replicates). (H) Comparison of laminin β4 signal intensity from G. (I) Laminin β4 levels in the ECM of severe FD SNs. ECM deposited by iPSC-FD-S2 and iPSC-ctr-C1 SNs was isolated on the indicated days and immunoblotted for laminin β4. Plates were coated with laminin β1, which was used as a loading control. (J) Signal intensity of blots from I (*n*=4 biological replicates). (C) One-way ANOVA followed by Tukey's multiple comparisons test. (E,J) Two-way ANOVA followed by Šídák's multiple comparisons test. (H) Two-way ANOVA followed by Tukey's multiple comparisons tests. ns, non-significant, *P<0.05, **P<0.01, ***P<0.001, ****P<0.0001. Data are mean±s.e.m. (A) Created in BioRender by Zeltner, N. (2006). https://BioRender.com/oo48aqb. This figure was sublicensed under CC-BY 4.0 terms.

So far, our results suggest that the ECM, particularly *LAMB4*, is crucial for SN development. Since two *LAMB4* single-nucleotide variants have been identified in individuals with severe FD symptoms (Zeltner et al., 2016) (Fig. 6A), we decided to investigate their impact on *LAMB4* expression. We used three iPSC lines from severe FD patients previously characterized (Zeltner et al., 2016): iPSC-FD-S1, iPSC-FD-S2 and iPSC-FD-S3 (Table S1). As controls, we used a healthy iPSC line (iPSC-ctr-C1) and a mild-FD iPSC line (iPSC-FD-M2), both of which do not harbor *LAMB4* variants. We first measured *LAMB4* expression during development. Similarly to control hPSC-ctr-H9 cells, iPSC-ctr-C1 and iPSC-FD-M2 cells (Saito-Diaz et al., 2024; Zeltner et al., 2016) expressed *LAMB4* starting at the late stages of NCC differentiation and the early stages of SN specification (Fig. 6B). However, *LAMB4* expression peaked on day 16, instead of day 20 in hPSC-ctr-H9 (Fig. 1C), possibly due to intrinsic differences between iPSCs and human embryonic stem cells (hPSC-ctr-H9). Interestingly, the three severe FD lines showed lower *LAMB4* expression compared to the controls (Fig. 6B,C) which was reflected at the protein level (Fig. 6D,E). In contrast to hPSC-ctr-H9 SNs, laminin β4 levels did not increase in the severe FD SNs, suggesting that the variants observed in these lines affect *LAMB4* transcription and translation (Fig. 6D,E). We confirmed these observations by immunofluorescence (Fig. 6F,G), where the signal intensity of laminin β4 in iPSC-ctr-C1 and iPSC-FD-M2 SNs was higher compared to iPSC-FD-S2 SNs (Fig. 6H). Finally, we measured the levels of laminin β4 in the ECM and confirmed that iPSC-FD-S2 SNs expressed lower laminin β4 levels compared to iPSC-ctr-C1 SNs (Fig. 6I,J).

We next asked whether restoring *ELP1* expression rescues *LAMB4* expression in FD. We used two previously characterized FD iPSC lines (iPSC-rescued-T6.1 and iPSC-rescued-T6.5) (Table S1) where the *ELP1* mutation was rescued (Zeltner et al., 2016). However, since they were generated from iPSC-FD-S2 cells, they still harbor the *LAMB4* variant identified in this cell line (Zeltner et al., 2016). Similar to the parental line (iPSC-FD-S2), iPSC-rescued-T6.1 and iPSC-rescued-T6.5 cells expressed low levels of *LAMB4* mRNA compared to iPSC-ctr-C1 cells (Fig. S5B,C). This was confirmed by immunoblotting of total laminin β4 and by immunofluorescence (Fig. S5D-F). Next, we tested whether *LAMB4* affects *ELP1* expression levels in iPSC-FD-S2*^LAMB4 OE dox^* and iPSC-FD-S3*^LAMB4 OE dox^*. We performed RT-qPCR and found no significant difference in *ELP1* expression upon *LAMB4* expression under doxycycline treatment in either cell line (Fig. S5G). Together, our results suggest that reduced *LAMB4* alongside an *ELP1* mutation, may contribute to severe FD symptoms with clinical implications. Moreover, *LAMB4* could serve as a marker for early detection of severe FD symptomatology in personalized medicine.

### Laminin β4 forms laminin-443 and regulates actin accumulation in sensory neurons *in vitro*

**Fig. 7.**
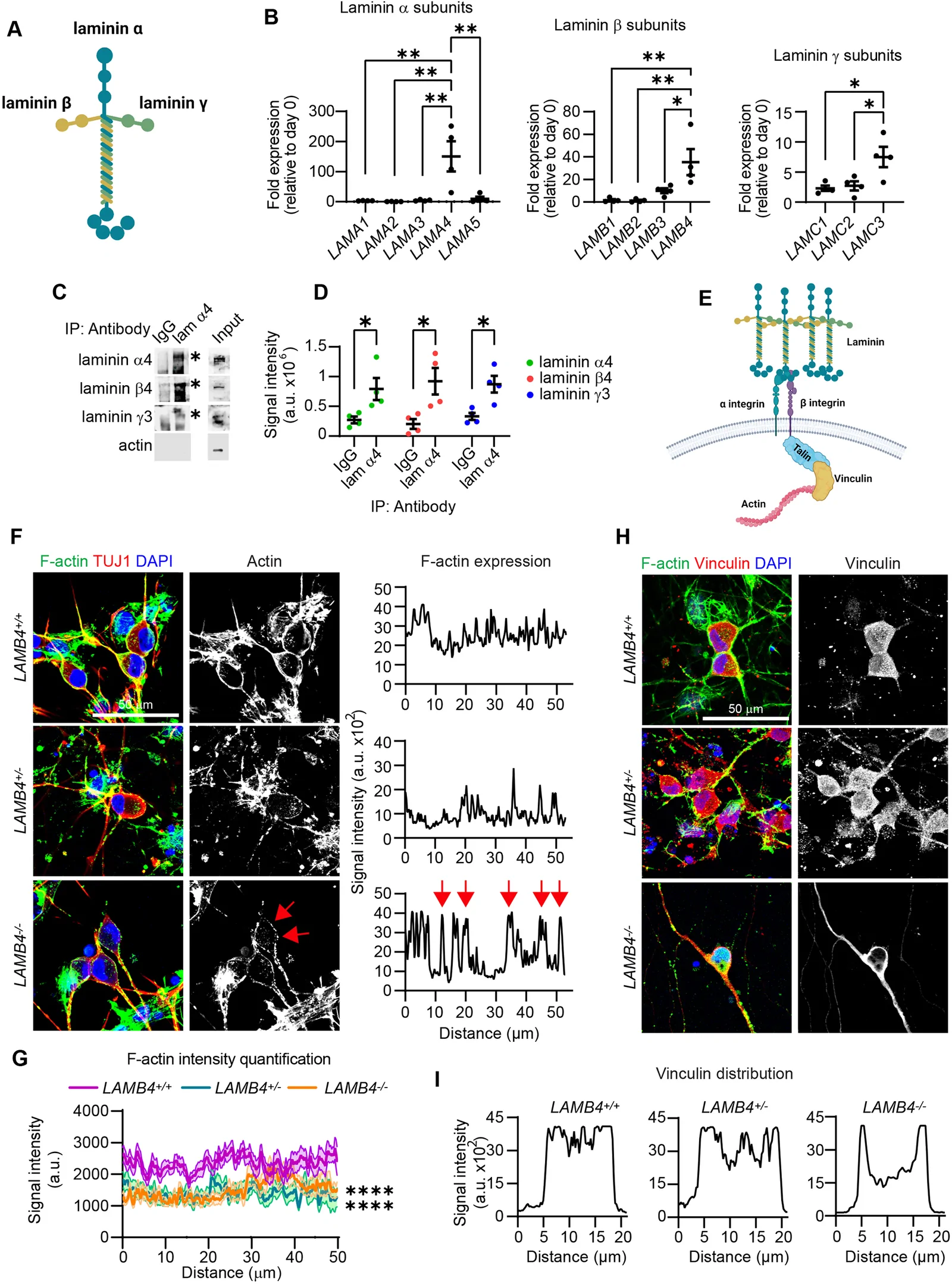
**Laminin β4 interacts with laminin α4 and laminin γ3, and regulates actin filament (F-actin) formation.** (A) Schematic of laminin trimer. (B) Expression of all laminin chains from day 16 hPSC-ctr-H9 SNs assessed by RT-qPCR (*n*=4 biological replicates). (C) Immunoprecipitation of laminin α4. Lysates from hPSC-ctr-H9 SNs were collected on day 30, followed by immunoprecipitation of laminin α4 (lam α4) and immunoblotting for laminin α4, laminin β4 and laminin γ3. Asterisks mark the band corresponding to each protein. (D) Quantification of signal intensity of immunoblots in C (*n*=4 biological replicates). (E) Schematic of intracellular pathways regulated by laminins. (F) Effects of *LAMB4* downregulation on F-actin. *LAMB4^+/+^*, *LAMB4^+/−^* and *LAMB4^−/−^* SNs were fixed on day 20 and stained for F-actin (phalloidin) and TUJ1, and using DAPI (left). A line was drawn around the cell body to measure F-actin signal intensity. A representative cell was plotted (right). Red arrows indicate signal from actin puncta. The distance refers to the length of the line used for measurements. (G) F-actin signal intensity of images from 20 cells in F (*n*=3 biological replicates). The distance is the length of the lines used for measurements. (H) Vinculin localization in *LAMB4* mutant SNs. Cells were fixed on day 20 and stained for F-actin (phalloidin) and vinculin, and using DAPI. (I) A line across the cell body was drawn and vinculin signal intensity was measured. A cell from a representative experiment from H was plotted. The distance refers to the length of the lines used for measurements. (B) One-way ANOVA followed by Tukey's multiple comparisons test. (D) Unpaired two-tailed *t*-test. (G) Two-way ANOVA followed by Tukey's multiple comparisons test. *P<0.05, **P<0.01, ****P<0.0001. Data are mean±s.e.m. (A,E) Created in BioRender by Zeltner, N. (2006). https://BioRender.com/oo48aqb. This figure was sublicensed under CC-BY 4.0 terms.

Given the role of *LAMB4*/laminin β4 in SN development and its clinical implications, we sought to further characterize its biology. Laminins are assembled in trimers consisting of α, β and γ chains (Domogatskaya et al., 2012) (Fig. 7A). Since no laminin β4-containing trimer has been described, we screened laminin chain expression in day 16 SNs and found *LAMA4* and *LAMC3* upregulated alongside *LAMB4* (Fig. 7B). *LAMA4* and *LAMC3* encode laminin α4 and γ3, respectively, suggesting laminin β4 forms part of laminin 443. In agreement with this, laminin α4 immunoprecipitated with laminin β4 and laminin γ3 (Fig. 7C,D). Next, we asked what the impact of laminin β4 was on SNs during development. Laminins bind to integrins at the plasma membrane that regulate the actin cytoskeleton via talin and vinculin (Fig. 7E), controlling cellular processes including cell migration (Bonnans et al., 2014). Based on our results showing that loss of *LAMB4* affects NCC migration, we hypothesized that laminin β4 also controls actin in SNs. We looked at the localization of F-actin using confocal microscopy in *LAMB4^+/+^*, *LAMB4^+/−^* and *LAMB4^−/−^* SNs. F-actin signal was evenly distributed around the cell body of *LAMB4^+/+^* SNs; however, the expression changed into a slightly punctate pattern in *LAMB4^+/−^* SNs which became more prominent in *LAMB4^−/−^* cells (Fig. 7F). This change also correlated with a decrease in F-actin signal intensity (Fig. 7G), suggesting that laminin β4 regulates the formation of F-actin in SNs. Vinculin localization was also *LAMB4* dependent (Fig. 7H,I). Vinculin was detected throughout the cytoplasm of *LAMB4^+/+^* SNs, possibly due to its localization at focal adhesions. In contrast, *LAMB4^+/−^* SNs showed vinculin accumulation at the plasma membrane and the cytoplasm, whereas vinculin was present mainly at the plasma membrane in *LAMB4^−/−^* SNs (Fig. 7H,I). These results show that laminin β4, via laminin-443, maintains F-actin at the cell body of SNs and transduces biophysical cues to the cell via vinculin. Without laminin β4, vinculin loses cytoplasmic localization and causes F-actin dissociation, impairing migration and differentiation. Our studies highlight a direct link between the cellular environment and intracellular components of SNs that could potentially be targeted to treat peripheral neuropathies.

## DISCUSSION

*LAMB4* (laminin β4) has been vastly understudied. We found *LAMB4* orthologs in many species but not in rodents (Fig. S1A). In addition, we show that *LAMB4* is expressed only in ectoderm lineages derived from NCCs and for a short period of time: (1) during the late stages of NCC migration and (2) in early stages of SN specification (Figs 1D and 2C). These factors make *in vivo* characterization of *LAMB4* (Fig. 2D,E) difficult.

*LAMB4* expression in late-stage NCCs suggests that it is important for the development of NCC-derived tissues. Our results showing that *LAMB4* downregulation reduces NCC migration (Fig. 3F-I) and a report showing that laminin β4 is expressed in the cutaneous basement membrane (Goletz et al., 2024) supports this hypothesis. This timing also explains our gene expression results, where *SOX10* expression does not change in *LAMB4^−/−^* NCCs. However, the reduction of *TFAP2A* suggests that laminin β4 influences the earliest stages of NCC specification at the neural plate border, consistent with *TFAP2A* and *SOX10* being regulated by partially independent gene regulatory networks during development (Hovland et al., 2020; Simões-Costa and Bronner, 2015). In contrast, genes expressed downstream of *SOX10* (*P75NTR*, *NGN1* and *NGN2*), which induce NCCs into SN lineages (Marmigère and Ernfors, 2007; Simões-Costa and Bronner, 2015), are downregulated. This is not unexpected, as other laminins also regulate NCC migration *in vivo* (Coles et al., 2006). In SNs, ECM accumulation of laminin β4 is visible over 20 days after mRNA is downregulated, suggesting that the spike of *LAMB4* transcription causes a burst of laminin β4 translation and secretion that is necessary to differentiate NCCs into SNs. Importantly, this transcriptional burst is not unique to our hPSC model. The re-analysis of the human fetal DRG snRNA-seq atlas revealed that *LAMB4* penetrance peaks in nociceptors by GW17 (Fig. 2C) before declining, and is sustained at lower levels in NCCs and SN progenitors between GW8 and GW12, further supporting the idea that a precisely timed burst of *LAMB4* expression is a conserved feature of human SN specification *in vivo*.

The ECM is required for multiple aspects of development, maturation, neuronal function (Melrose et al., 2021; Nishimune et al., 2004; Chelyshev et al., 2022) and synaptogenesis (Pyka et al., 2011). Moreover, the ECM provides the necessary cues required for axon elongation and guidance during development and after injury (Kubo et al., 2002; Melrose et al., 2021; Roumazeilles et al., 2018). We observed that axons of *LAMB4^−/−^* SNs show an irregular elongation pattern compared to control SNs (Fig. 4B), suggesting that laminin β4 regulates this process. These deficiencies in axon elongation could affect SN homeostasis and explain why *LAMB4^−/−^* SNs degenerate at high rates (Fig. 4I). This also suggests that laminin β4 secreted by differentiated SNs is required for their survival and agrees with the literature showing that neurons release laminins (Hagg et al., 1997; Nirwane and Yao, 2019; Omar et al., 2017; Pyka et al., 2011).

*LAMB4* downregulation is linked to diseases of the PNS, including peripheral neuropathies. Individuals with sporadic cases of peripheral neuropathy diverticulitis, caused by reduced density of NCC-derived enteric neurons, have also been shown to harbor *LAMB4* variants that result in its downregulation (Coble et al., 2017; Lake and Heuckeroth, 2013; Wedel et al., 2010). *LAMB4* has also been linked to FD-related peripheral neuropathy. Patients with severe but not with mild FD symptoms harbor mutations in *LAMB4* (Zeltner et al., 2016). Here, we show that these mutations downregulate *LAMB4* expression (Fig. 6D-H) and could explain the symptomatic differences between individuals with mild and severe symptoms. Moreover, our results using the ECM of healthy, mild and severe FD cells suggest a model where the ECM impacts the differentiation of NCCs and *LAMB4* is required to push them towards the SN fate. However, it will be important to test whether the presence of laminin β4 in the ECM is sufficient to rescue the severe FD phenotypes. Our results hint at the possibility that *LAMB4* could be used as a diagnostic marker for severe FD onset early in life.

Approximately 99.5% of FD patients have a mutation in *ELP1* (González-Duarte et al., 2023). ELP1 is the scaffold protein of the elongator complex, and it is involved in transcription and tRNA modification during translation (Nguyen et al., 2010). Our results show that restoring *ELP1* expression in severe FD iPSCs does not impact *LAMB4* levels, and mild but not severe FD SNs express high *LAMB4* levels*.* This suggests that *LAMB4* expression is not completely *ELP1* dependent. Another possibility is that ELP1 impacts laminin β4 translation due to defects in tRNA production. Thus, laminin β4 translation could be downregulated in mild FD SNs due to the *ELP1* mutation, whereas in severe FD SNs, *LAMB4* mRNA expression (due to the identified *LAMB4* mutations) and translation (due to the *ELP1* mutation) are both affected. Further studies will be necessary to dissect this mechanism.

We show that laminin β4 is part of laminin-443. Laminin α4 is expressed in the DRG (where NCCs develop into SNs) during mouse development (Miner et al., 1997), which strengthens the hypothesis that laminin β4 is involved in SN development. However, because *LAMB4* is not expressed in rodents (Fig. 2D,E), it would be necessary to confirm this possibility in other *in vivo* models. Laminins bind to integrins located on the cell surface and connect to the actin cytoskeleton (Bouvard et al., 2013). Laminin α4 has been shown to interact with integrins α3β1 and α6β1 (Fujiwara et al., 2001; Pang et al., 2023); however, this interaction has not been explored in SNs. Our results show that *LAMB4* loss reduces F-actin formation and changes vinculin localization, possibly due to integrin inactivation. Vinculin is a cytoplasmic protein that links integrins to actin and translates biophysical cues from the ECM into intracellular biochemical signals (Atherton et al., 2016). Upon integrin activation, vinculin is recruited to F-actin and focal adhesions (Atherton et al., 2016; Bouvard et al., 2013; Byron et al., 2015). It is possible that, in *LAMB4^−/−^* SNs, integrins are inactivated and vinculin associates with other interactors at the cell membrane (Bakolitsa et al., 1999). These are interesting observations, as most studies of actin in neurons focus on the growth cone (Omotade et al., 2017) and open new avenues for studying the cell biology of peripheral neurons. Furthermore, understanding ECM and actin regulation in the PNS will uncover new mechanisms that could be used to promote neuronal regeneration and treat peripheral neuropathies.

## MATERIALS AND METHODS

### hPSC maintenance

hPSC-ctr-H9 human embryonic stem cells (WA-09, WiCell) and all human induced pluripotent stem cells were grown at 37°C with 5% CO_2_ in vitronectin-coated dishes (ThermoFisher, A31804, 5 μg/ml, 1 h at room temperature). Cells were fed daily with Essential 8 Medium+Supplement (Gibco, A1517001). Cells were split at a 1:10 ratio using the following protocol: cells were washed with PBS, incubated with 0.5 mM EDTA and 3.08 M NaCl in PBS for 2 min at 37°C, and then resuspended in E8+Supplement. iPSC-ctr-C1, iPSC-FD-M2, iPSC-FD-S1, iPSC-FD-S2 and iPSC-FD-S3 have been previously characterized (Zeltner et al., 2016).

### Sensory neuron differentiation

Differentiation was done as previously described (Saito-Diaz and Zeltner, 2022; Saito-Diaz et al., 2021). Prior to differentiation, plates were coated with vitronectin (5 μg/ml) and incubated for 1 h at room temperature. On day of plating (day 0), hPSCs were washed with PBS, incubated with 0.5 mM EDTA and 3.08 M NaCl in PBS for 20 min, and plated at a density of 200,000 cells/cm^2^ in NC differentiation media (day 0-1) containing: Essential 6 Medium (Gibco, A1516401), 10 μM SB431542 (R&D Systems, 1614), 1 ng/ml BMP4 (R&D Systems, 314-BP), 300 nM CHIR99021 (R&D Systems, 4423) and 10 μM Y-27632 (Biogems, 1293823). BMP4 concentration was titrated for each line. Accordingly, BMP4 was not used with iPSC-FD-S1 and iPSC-FD-S3 cells. The next day, the cells were fed with NC differentiation media (day 0-1). From day 2 to 12, cells were fed every 2 days with NC differentiation media (day 2-12) containing: Essential 6 Medium, 10 μM SB431542, 0.75 μM CHIR99021, 2.5 μM SU-5402 (Biogems, 2159233) and 2.5 μM DAPT (R&D Systems, 2634).

On day 10, plates were coated with 15 μg/ml poly-L-ornithine (PO, Sigma, P3655) in PBS and incubated at 37°C overnight. On day 11, the plates were washed three times with PBS and coated with 2 μg/ml laminin-1 (LM, Cultrex, 3401-010-02) and 2 μg/ml human fibronectin (FN, Corning, 47743-654) in PBS and incubated overnight. On day 12, cells were resuspended using Accutase (Innovative Cell Technologies, NC9464543) for 20 min, washed with PBS and resuspended in SN Media containing Neurobasal media (Gibco, 21103-049) containing 1× N2 (Gibco, 17502-048), 1× B-27 (Gibco, 12587-010), 2 mM L-glutamine (ThermoFisher, 25030-081), 20 ng/ml GDNF (Peprotech, 450-10), 20 ng/ml BDNF (R&D Systems, 248-BD), 25 ng/ml NGF (Peprotech, 450-01), 600 ng/ml of laminin-1, 600 ng/ml fibronectin, 1 μM DAPT and 0.125 μM retinoic acid (Sigma, R2625). Cells were then replated at a density of 250,000 cells/cm^2^ onto PO/LM/FN-coated plates. For coating and cell culture, we used murine laminin-1 (Laminin-111), which is composed of α1, β1 and γ1 chains. While exogenous LM111 provides a general adhesion substrate, it does not contain the β4 chain and is therefore unlikely to compensate for *LAMB4* loss or confound the endogenous functions of laminin β4 examined in this study. The media was replaced the following day. Cells were fed every 2-3 days. On day 20, DAPT was removed. For *LAMB4* overexpressed cell lines, *LAMB4^−/− OE +dox^*, *LAMB4^−/− OE −dox^* iPSC-FD-S2 *^LAMB4 OE −dox^*, iPSC-FD-S2 *^LAMB4 OE +dox^*, iPSC-FD-S3 *^LAMB4 OE −dox^* and iPSC-FD-S3 *^LAMB4 OE +dox,^* we started adding doxycycline 1 μg/ml (Sigma, 24390-14-5) from day 2 onwards with every media change. Differentiation progress was followed using a bright-field microscope (Leica).

### Endoderm differentiation

Endoderm differentiation was performed as described previously (Holloway et al., 2020; Zeltner et al., 2016). On day 0, hPSC-ctr-H9 cells were washed with PBS and incubated with Accutase for 20 min and seeded at a density of 100,000 cells/cm^2^ in RPMI medium (ThermoFisher, 12633012) with Glutamax (ThermoFisher, 35050061) and 100 ng/ml Activin A (R&D Systems, 338-AC-010). Cells were fed daily for 3 days and FBS was added at increasing concentrations: 0%, 0.2% and 2%.

### Mesoderm and cardiomyocyte differentiation

Cardiomyocyte differentiation was carried out as previously described (Wang et al., 2021). hPSC-ctr-H9 colonies were washed with PBS followed by incubation with Accutase for 20 min. Cells were resuspended in E8 medium+supplement and seeded at a density of 100,000 cells/cm^2^. When the cells reached ∼80% confluency, the cells were fed with RPMI medium supplemented with insulin-free B27 (ThermoFisher, A1895601) and 6 μM CHIR99021 for 2 days. One day later, the medium was replaced with RPMI+insulin-free B27. On day 4, cells were fed with RPMI+insulin-free B27 with 5 μM IWP2 (Cayman Chemical, 13951). The following day, the medium was replaced with RPMI+insulin-free B27. The cells were fed on day 7 with RPMI+insulin-free B27 and medium was replaced every 2 days.

### Plasmid construction

The human *LAMB4* coding sequence (NM_007356) was PCR-amplified from a commercially obtained tagged ORF clone (OriGene, RC215486) using the following primers: forward, 5′-GGCACCAAAATCAACGGGAC-3′; reverse, 5′-TGACTCTCGAGTCAGCTATAGCACCTAGCATATTTTTTTTCTTGT-3′. The amplified product was cloned into the pENTR entry vector and verified by Sanger sequencing. The *LAMB4* insert was placed into the doxycycline-inducible lentiviral destination vector pInducer20 (Addgene #44012) via Gateway LR recombination (Thermo Fisher Scientific) for lentivirus production.

### Lentivirus production

Lentiviral vectors were transfected into HEK293T cells with packaging vectors in the presence of polyethylenimine (Polysciences). Viral supernatants were collected 72 h after transfection, and viral particles were concentrated by ultracentrifugation at 100,000 ***g*** for 1.5 h at 4°C.

### RNA isolation and RT-qPCR

RNA was isolated using Trizol (ThermoFisher, 15596026) according to the manufacturer's conditions and resuspended in 20 μl RNase-free water. RNA concentration and purity was measured using NanoDrop One (ThermoFisher). 1 μg of RNA was converted to cDNA using iScript cDNA Synthesis kit (BioRad, 1708841) according to the manufacturer's instructions and diluted 1:100 in RNase-free water. RT-qPCR reactions were run with 1 µl of cDNA and SYBR Green Supermix (BioRad, 1725272) according to the manufacturer's conditions in a C1000 Touch Thermal Cycler CFX96 (BioRad). The following cycling parameters were used: 95°C for 5 min, 40 cycles of 95°C for 5 s and 60°C for 10 s. Results were analyzed using the comparative CT method. GAPDH was used as a housekeeping gene. The sequences of primers used in this study are available in Table S2.

### Antibodies

The following antibodies were used: anti-laminin β4 (Abcam, ab150819; Sigma, HPA020242), anti-laminin β1 (Abcam, ab44941), anti-laminin α4 (R&D Systems, AF7340), anti-laminin γ3 (Proteintech, 67261-1-I), anti-SOX10 (Santa Cruz, sc-365692), anti-TFAP2A (Abcam, ab108311), anti-BRN3A (Millipore, MAB1585), anti-TUJ1 (Biolegend, 801201), anti-ISL1 (DSHB, 39.4D5-c), anti-PRPH (Santa Cruz, sc-377093), anti-Actin (BD Biosciences, 612656), anti-Vinculin (Abclonal, A14193), anti-αSMA (Sigma, A5228), anti-Phalloidin-iFluor 488 (Abcam, ab176753), anti-CD49d-PE/Cy7 (Biolegend, 304314), anti-GAPDH (Cell Signaling Technology, 2118S), anti-TRKA-PE (R&D Systems, FAB1751P), anti-TRKB-AF647 (R&D Systems, FAB3971R), anti-TRKC-PE (R&D Systems, FAB373P), anti-cleaved caspase 3 (Cell Signaling Technology, 9661) and anti-HA-Tag (Abcam, ab9110). The following secondary antibodies were used: goat anti-mouse IgG1 AF488 (ThermoFisher Scientific, A21121), goat anti-mouse IgG2a (ThermoFisher Scientific, A-21131), goat anti-mouse IgG2b (ThermoFisher Scientific, A21242), donkey anti-rabbit AF647 (ThermoFisher Scientific, A31573), donkey anti-mouse AF488 (ThermoFisher Scientific, A21202), goat anti-mouse HRP (ThermoFisher Scientific, 62-6520), goat anti-rabbit HRP (ThermoFisher Scientific, 65-6120), goat anti-rat HRP (ThermoFisher Scientific, A18865) and donkey anti-sheep HRP antibody (Jackson Immunoresearch, 713-035-003). The dilutions used are indicated in the relevant methods section.

### Immunoblotting

To collect cell lysates, cells differentiated in 6-well plates were washed with PBS and incubated with 120 μl of RIPA buffer (Sigma, R0278) with 1 mM PMSF and 1× PhosSTOP (Roche, 906845001) for 15 min on ice. Cells were then scrapped and the lysate transferred to an Eppendorf tube, followed by mixing for 10 s using a vortex and centrifuged at 13,500 ***g*** for 10 min at 4°C. Supernatants were transferred to a new Eppendorf tube and protein concentration was measured. Samples were mixed with 2× Laemmli buffer containing β-mercaptoethanol and run in 7.5% polyacrylamide gels under denaturing conditions using MOPS buffer at 130 V. Proteins were transferred to a nitrocellulose membrane and blocked for 30 min in 5% non-fat dry milk in 0.1% TBS-T (0.1% Tween-20, 50 mM Tris-HCl and 150 mM NaCl at pH7.6). Primary antibodies were added to the membranes in blocking buffer (anti-laminin β4, 1:1000; anti-laminin α4, 1:1000; anti-laminin γ3, 1:1000; anti-Actin, 1:5000) and incubated overnight at 4°C. Blots were then washed three times with 0.1% TBS-T and incubated with goat anti-mouse HRP, goat anti-rabbit HRP, goat anti-rat HRP or donkey anti-sheep HRP antibody (1:5000) for 1 h at room temperature. Blots were washed three times with 0.1% TBS-T and incubated with Clarity Western ECL Substrate (BioRad, 1705061). Chemiluminescence signal was detected using UVP ChemStudio (Analytic Jena). Signal quantification was carried out using Image Studio Lite (LICOR).

### Immunoprecipitation

Lysates were collected and concentration was measured as described above. Magnetic protein A/G beads (25 μl, ThermoFisher, 88802) were pre-washed three times with RIPA buffer with 1 mM PMSF and 1× PhosphoSTOP and incubated with 1 μg of laminin α4 antibody for 30 min at 4°C in a rotator. Beads were then washed three times with RIPA buffer with 1 mM PMSF and 1× PhosphoSTOP and incubated overnight with 1 mg of lysate. The following day, beads were washed three times with RIPA buffer with 1 mM PMSF and 1× PhosphoSTOP and resuspended in 2× Laemmli buffer.

### Immunofluorescence

NCCs and SNs differentiated in 24- or 4-well plates were washed once with PBS and fixed with 4% paraformaldehyde (ThermoFisher, AAJ19943K2) for 20 min at room temperature. Cells were then washed with PBS and incubated for 20 min with permeabilization buffer containing 1% BSA, 0.3% Triton-X, 3% goat or donkey serum and 0.01% sodium azide in PBS. Cells were then incubated with the indicated primary antibodies (anti-laminin β4, 1:100; anti-SOX10, 1:100; anti-TFAP2A, 1:500; anti-BRN3A, 1:100; anti-TUJ1, 1:1500; anti-ISL1, 1:200; anti-PRPH, 1:100; anti-αSMA, 1:100) in antibody buffer containing 1% BSA, 3% goat or donkey serum and 0.01% sodium azide overnight at 4°C. Cells were then washed three times in PBS and incubated with secondary antibodies in antibody buffer for 1 h. Cells were washed with PBS, incubated with DAPI (1:1000) for 5 min, washed with PBS and stored at 4°C. Imaging was carried out using a Lionheart FX fluorescence microscope (BioTek). Image analyses and quantifications were carried out in Fiji. For quantifications, five different fields were imaged and quantified. For confocal microscopy, 50,000 NCCs were seeded in PO/LM/FN-coated 4-well chamber slides (iBidi, 80426) on day 12. On day 20, SNs were fixed and stained as described above. Primary antibodies used were: anti-TUJ1 (1:1500) and anti-Vinculin (1:100). Phalloidin-iFluor 488 (1:1000) was incubated with secondary antibodies for 1 h. Imaging was carried out using an Olympus FV1200 Confocal Laser Scanning Microscope using Argon and Helium-Neon lasers. Images were taken as *z*-stacks of 3 μm of height. ImageJ was used to obtain maximum intensity projections and to measure the signal intensity profiles.

### Flow cytometry

On the indicated days, cells were washed with PBS and incubated with Accutase for 30 min at 37°C. Cells were then washed and resuspended in flow buffer (DMEM, 2% FBS, and 1 mM L-glutamine) followed by centrifugation at 200 ***g*** for 4 min. Cells were resuspended in ice-cold PBS, counted and diluted to a concentration of 1×10^6^ cells/100 μl. For NCCs, cells were centrifuged at 200 ***g*** for 4 min at 4°C and resuspended in 100 μl of Flow buffer and incubated with anti-CD49d-PE/Cy7 antibody (1:160) for 30 min, or with anti-TRKA-PE (1:20), anti-TRKB-AF647 (1:20) or anti-TKC-PE (1:20) antibodies for 1 h on ice. Samples were washed twice with flow buffer, resuspended in 300 μl of flow buffer with DAPI (1:1000), filtered and analyzed using a Cytoflex S (Beckman Coulter). For SNs, cells at a concentration of 1×10^6^ cells/100 μl were centrifuged, resuspended in 300 μl BD Cytofix buffer (BD Biosciences, 554655) and incubated on ice for 30 min. Cells were centrifuged for 4 min at 380 ***g*** and resuspended in 600 μl of cold BD perm/wash buffer (BD Biosciences, 554723). Goat serum (30 μl) was added to the cells and incubated on ice for 30 min. Cells were divided into three tubes (200 μl each): (1) unstained control, (2) secondary antibody control and (3) sample. All tubes were centrifuged for 4 min at 2000 RPM and the cells were resuspended in 200 μl of antibody buffer (BD perm/wash buffer+10 μl goat serum) with or without anti-BRN3A antibody (1:100) and incubated overnight at 4°C. Cells were then washed twice with 300 μl BD Perm/Wash buffer, resuspended in antibody buffer with or without AF488 goat-anti-mouse (1:500) and incubated on ice for 30 min. Cells were then washed three times with BD perm/wash buffer, filtered and analyzed using a Cytoflex S (Beckman Coulter). Analyses were carried out using FlowJo.

### Scratch assay

On day 8, NCCs differentiated from *LAMB4^+/+^*, *LAMB4^+/−^* and *LAMB4^−/−^* hPSCs were washed with PBS and incubated with Accutase for 20 min at 37°C. Cells were resuspended in NC differentiation media (day 2-12), counted and replated at a density of 60,000 cells/cm^2^ in 4-well or 24-well plates. When the cells reached confluency, a scratch was performed in the center of the well using a 1000 μl sterile tip. Bright-field images were immediately taken (0 h) was taken using a Lionheart FX (Bio-Tek) fluorescent microscope. Subsequent images were taken 24 and 48 h later at the same coordinates. Images were analyzed as previously described (Pijuan et al., 2019).

### Live-cell imaging

On day 8, NCCs from *LAMB4^+/+^*, *LAMB4^+/−^* and *LAMB4^−/−^* hPSCs were washed with PBS, incubated with Accutase for 20 min at 37°C and resuspended in NC differentiation media (day 2-12). Cells were then counted and replated at a density of 15,000 cells/cm^2^ in 4-well or 24-well plates. Medium was replaced the following day and bright-field images were taken every 10 min for 18 h using a Lionheart FX microscope (Bio-Tek) with climate control chamber. Cells were maintained at 37°C with 5% CO_2_ throughout the experiment. Each experiment was performed in triplicate (technical replicate) and ∼60-80 cells were tracked per well. Individual images were compiled using Fiji and individual cells were tracked using TrackMate (v7.13.2) (Ershov et al., 2022; Tinevez et al., 2017). Tracks of individual cells were exported and analyzed using the Chemotaxis and Migration Tool software (Ibidi).

### Generation of *LAMB4* mutant hPSCs

Two gRNAs (GCTCAAGATGACTGCAACAG and CTGGTGATCTCCTGGTGGGC) targeting exon 3 of *LAMB4* were selected using E-CRISP (Heigwer et al., 2014) (available at www.E-CRISP.org). The oligos were annealed, phosphorylated and ligated into PX458 using T4 DNA ligase. The resulting plasmid was transformed into DH5α bacteria and colonies were screened by Sanger sequencing. The resulting plasmids (PX458-LAMB4gRNA1 and PX458-LAMB4gRNA2) were transfected into hPSC-ctr-H9 cells using Lipofectamine Stem Transfection Reagent (ThermoFisher, STEM00001) following to the manufacturer's protocol. After 48 h, cells were washed with PBS and incubated with Accutase for 20 min at 37°C. The cells were transferred to a 15 ml conical tube, filled with PBS and centrifuged at 200 ***g*** for 5 min. The supernantant was aspirated and the pellet was resuspended in sorting medium containing Essential 8 Medium+Supplement, 1× CloneR (Stemcell Technologies, 05889) and 10 μM Y-27632. Cells were then counted and 2×10^6^ cells were transferred to an Eppendorf tube and resuspended in 400 μl of sorting medium containing 0.4 μl of Propidium Iodide (ThermoFisher, P3566). The resuspended cells were filtered using a round-bottom FACS tube and GFP^+^ cells were sorted using a FACS Melody Cell Sorter System (BD Biosciences). Individual cells were sorted to VTN-coated 96-well plates with prewarmed 50 μl of sorting medium in each well. The cells were fed every 24 h for ∼10 days. When colonies started to emerge, cells were transferred to 24-well plates using EDTA and the protocol previously described. Genomic DNA was isolated from each clone and screened. Positive clones were further expanded.

### Electrophysiology experiments

Experiments were performed using a Maestro Pro (Axion Biosystems) multi-electrode array (MEA) system. On day 12, NCCs were seeded (250,000 cell/cm^2^) onto PO/LM/FN-coated BioCircuit MEA 96 plates (Axion Biosystems, cat# M768-BIO-96) containing 8 embedded electrodes/well, in SN Media as previously described, and allowed to continue differentiating. Recordings were made every 2-3 days at 37°C with a sampling frequency of 12.5 kHz for 5 min. Recordings from at least 6 wells per reading were averaged. Firing frequency was normalized to the number of active electrodes. Bursts were detected using Inter-Spike Interval. Capsaicin (Sigma, cM2028) and WIN 55,212-2 (R&D Systems, 1038) were resuspended in DMSO and added to the cells 3 min prior to starting recordings. Hypoosmotic media was obtained by mixing SN Media with sterile water in a 45:55 ratio and it was added to the cells prior to recordings.

### Degeneration assay

On day 12, NCCs from *LAMB4^+/+^*, *LAMB4^+/−^* and *LAMB4^−/−^* hPSCs were replated on 4-well plates (ThermoScientific, 12-565-72), at 250,000 cells/cm^2^, coated with PO/FN in SN media with 1 ng/ml NGF. Cells were fed every 2-3 days. DAPT was removed after day 20. Cells were fixed on day 13, 20, 27 and 34 and stained for BRN3A and TUJ1.

### Extracellular matrix isolation and rescue experiments

NC- and SN-derived ECM was isolated as previously described (Hellewell et al., 2017). To isolate ECM from SNs, day 12 hPSC-ctr-H9, iPSC-ctr-C1 and iPSC-FD-S2 NCCs were resuspended in Accutase, as described above, and seeded in 60 mm dishes. On day 30, cells were washed with 3 ml of PBS and incubated with 20 mM ammonium hydroxide (Sigma, 221228-100ML-A). The dishes were constantly shaken for 5 min at room temperature, followed by 5 washes with 5 ml of de-ionized water. For immunoblotting, the ECM was scrapped and resuspended in Laemmli buffer containing β-mercaptoethanol and 100 mM dithiothreitol (DTT, RPI, D11000) preheated at 95°C for 2 min. For ECM rescue experiments, hPSC-ctr-H9, iPSC-FD-M2 and iPSC-FD-S3 were differentiated using the SN differentiation protocol described above. On day 12 (NCCs) and day 30 (SNs) the cells were treated following the ECM isolation protocol. The undisturbed ECM was kept in the plates in de-ionized water. To start the differentiation, water was aspirated and iPSC-FD-S3 cells were seeded following the SN differentiation protocol described above.

### RNAseq and phylogenetic analyses

RNAseq data from endoderm (Loh et al., 2014) (GSE52658) and mesoderm (Loh et al., 2016) (GSE85066) were analyzed. FPKM and TPM results were converted to log2 and graphed as heatmaps. For laminin chains analysis, the following sequences from NCBI were used: LAMB1: *D. rerio* (NP_775382), *X. tropicalis* (XP_002933140), *M. musculus* (XP_006515056), *R. norvegicus* (XP_003750185), *C. lupus* (XP_038279702), *B. taurus* (NP_001193448), *M. mulatta* (XP_014990159), *H. sapiens* (XP_047276315), *P. troglodytes* (XP_001165667), *G. gallus* (XP_046780211), *A. carolinensis* (XP_016849500), *S. purpuratus* (XP_030828530), *D. melanogaster* (NP_476618), *A. mellifera* (XP_006571829), *C. elegans* (NP_500734); (2) LAMB2: *M. musculus* (NP_001398157), *R. norvegicus* (XP_006243771), *M. mulatta* (XP_014986301), *H. sapiens* (XP_005265184), *P. troglodytes* (XP_016796574), *C. lupus* (XP_038283703), *B. taurus* (XP_010816035), *G. gallus* (NP_989497), *A. carolinensis* (XP_062829843), *D. rerio* (XP_005162102), *X. tropicalis* (XP_004914156), *D. melanogaster* (NP_524006); (3) LAMB3: *D. rerio* (XP_700808.6), *G. gallus* (XP_040547616), *X. tropicalis* (XP_012826649), *A. carolinensis* (XP_062834708), *M. musculus* (XP_006497296), *R. norvegicus (*XP_008768078), *B. taurus* (XP_005217424), *C. lupus* (XP_038526808), *M. mulatta* (XP_014973102), *H. sapiens* (XP_005273181), *P. troglodytes* (XP_054514183); (4) LAMB4: *D. rerio* (XP_068073408), *X. tropicalis* (XP_031754867), *C. lupus* (XP_038310194), *M. mulatta* (XP_028702003), *H. sapiens* (XP_011514277), *P. troglodytes* (XP_063672018), *G. gallus* (XP_040515061) and *A. carolinensis* (XP_062837767). Alignments were made using Clustal Omega (Madeira et al., 2024) using default settings. The phylogenetic tree was visualized using Treeviewer.

### Human embryonic DRG snRNA-seq

Data were obtained from Lu et al. (2024) (GSE245310), spanning gestational weeks (GW) 7-21 and comprising 98,323 cells. Raw expression matrices were downloaded from GEO and imported into AnnData objects in Python 3.10. Genes detected in fewer than three cells were removed,; cells with fewer than 200 detected genes were excluded. Counts were normalized to a total of 10,000 per cell and log-transformed using log1p; the log-normalized matrix was preserved before feature scaling and used for all downstream expression visualizations. Highly variable genes were identified with Scanpy (n=2500), followed by principal component analysis (PCA; 40 components) and construction of a k-nearest-neighbor graph with 30 neighbors per cell. A uniform manifold approximation and projection (UMAP) embedding was computed from the PCA space using a minimum distance of 0.3 and a fixed random seed (42), yielding a reproducible two-dimensional representation of the global human fetal DRG manifold. No additional batch correction was applied, consistent with the analytical approach taken by Lu et al. (2024), in which inter-sample variation was found to reflect developmental stage rather than technical batch effects. Inter-sample clustering was assessed by visualizing UMAP colored by sample identity, confirming that cells from different gestational weeks intermixed within biologically coherent clusters.

Cell types were annotated by marker-gene scoring using canonical signatures from Lu et al. Briefly, we defined gene sets for neural crest cells (*SOX10* and *PAX3*), sensory neuron progenitors (*NEUROG1*, *NEUROG2*, *SIX1*, *EYA1*, *EYA2*, *ISL1*, *RBFOX3*, *POU4F1* and *ELAVL3*), nociceptors (*NTRK1*, *RUNX1*, *TRPA1*, *SCN10A* and *TRPM8*), mechanoreceptors (*NTRK2* and *RET*), proprioceptors (*NTRK3* and *RUNX3*), TH+ neurons (*TH*), Schwann cell progenitors (*SOX2* and *FOXD3*), Schwann cells (*MBP*, *MAG*, *MPZ* and *PLLP*), satellite glia (*FABP7*, *APOE*, *GJA1, SLC1A3* and *S100B*), endothelial cells (*PECAM1* and *EMCN*), vascular smooth muscle cells (*TPM2* and *ACTA2*), muscle cells (*PAX7* and *MYH3*), macrophages (*MRC1*), and red blood cells (*HBA1*). For each gene set, we computed a module score, assembled the scores into a matrix and assigned each cell to the label with the highest score; cells with no positive score were classified as ‘Unknown’; red blood cells were excluded from all downstream visualizations.

Gestational week metadata were extracted directly from cell barcodes by regular-expression matching of “GWxx” substrings and stored as a categorical variable. For developmental trajectory analyses, this categorical label was converted to a numeric gestational age axis (GW7-GW21) by parsing the embedded integers. The sensory neuron lineage was defined *a priori* as neural crest cells, sensory neuron progenitors, nociceptors, mechanoreceptors, proprioceptors, TH+ neurons and C-LTMRs. TH+ neurons and C-LTMRs were excluded from developmental trajectory analyses because these populations contained fewer than three cells per gestational week for most timepoints (minimum *n* required=3), precluding robust longitudinal estimation.

*LAMB4* expression was retrieved from the raw (pre-scaled) expression matrix when available; if *LAMB4* was not present, we used the corresponding column from the log-normalized matrix. Values were clipped at a minimum of zero, and the 98th percentile of *LAMB4*-positive cells (p98) was used as the upper limit of the color scale to reduce the visual impact of rare extreme outlier cells. For UMAP overlays, *LAMB4*-negative cells were rendered in light grey, while *LAMB4*-positive cells were plotted in ascending order of expression using a continuous white-to-maroon colormap, thereby enhancing the visibility of low-to-moderate expression within sparsely positive populations.

### Mouse embryonic DRG scRNA-seq

Data (E9.5-E12.5) were obtained from Faure et al. (2020) (GSE150150) as a processed loom file. UMAP coordinates were aligned by matching cell barcodes between the loom object and the published coordinate table after removal of dataset-specific suffixes. Gene identifiers were converted from Ensembl IDs to gene symbols by identifying the variable metadata column containing canonical developmental markers (*SOX10*, *NTRK1*, *RUNX1*, *PVALB*, *MPZ* and *TUBB3*). We computed PCA, constructed a neighbor graph, and performed Leiden clustering, subsequently annotating each cluster using the identical marker-gene logic applied to the human dataset. Expression of the laminin β family (*LAMB1*, *LAMB2*, *LAMB3* and *LAMB4*) was queried in the normalized expression matrix. While *LAMB1*, *LAMB2* and *LAMB3* were detected, all cells exhibited *LAMB4* expression equal to zero, indicating an absence of detectable *LAMB4* transcripts across neural and glial lineages within this developmental window.

### Statistical analysis

All analyses and graphs were carried out using PRISM (GraphPad). Statistical analyses are indicated in each figure legends. An unpaired two-tailed Student's *t*-test was used to compare two groups. One-way analysis of variance (ANOVA) followed by Dunnett's or Tukey's multiple comparisons test was used to compare three or more groups. Two-way ANOVA followed by Šídák's multiple comparisons test was used to analyze data sets with two variables. Data are shown as mean±s.e.m. In all experiments, the differences were considered significant when *P*<0.05. The numbers of biological replicates (*n*) are defined as the number of independent differentiations started at least 3 days apart or from a different vial of cells. The numbers of biological replicates are indicated in the figure legends.

## Supplementary Material

10.1242/develop.205371_sup1 Supplementary information
