# Supplementary material for: Laminin β4 is required for the development of human peripheral sensory neurons
Source: Development. 2026 Sep 3;153(16):dev205371. doi: 10.1242/dev.205371 (PMC13615688; doi:10.1242/dev.205371)
Supplement: Supplementary file 1 [file develop-153-205371-s1.pdf]

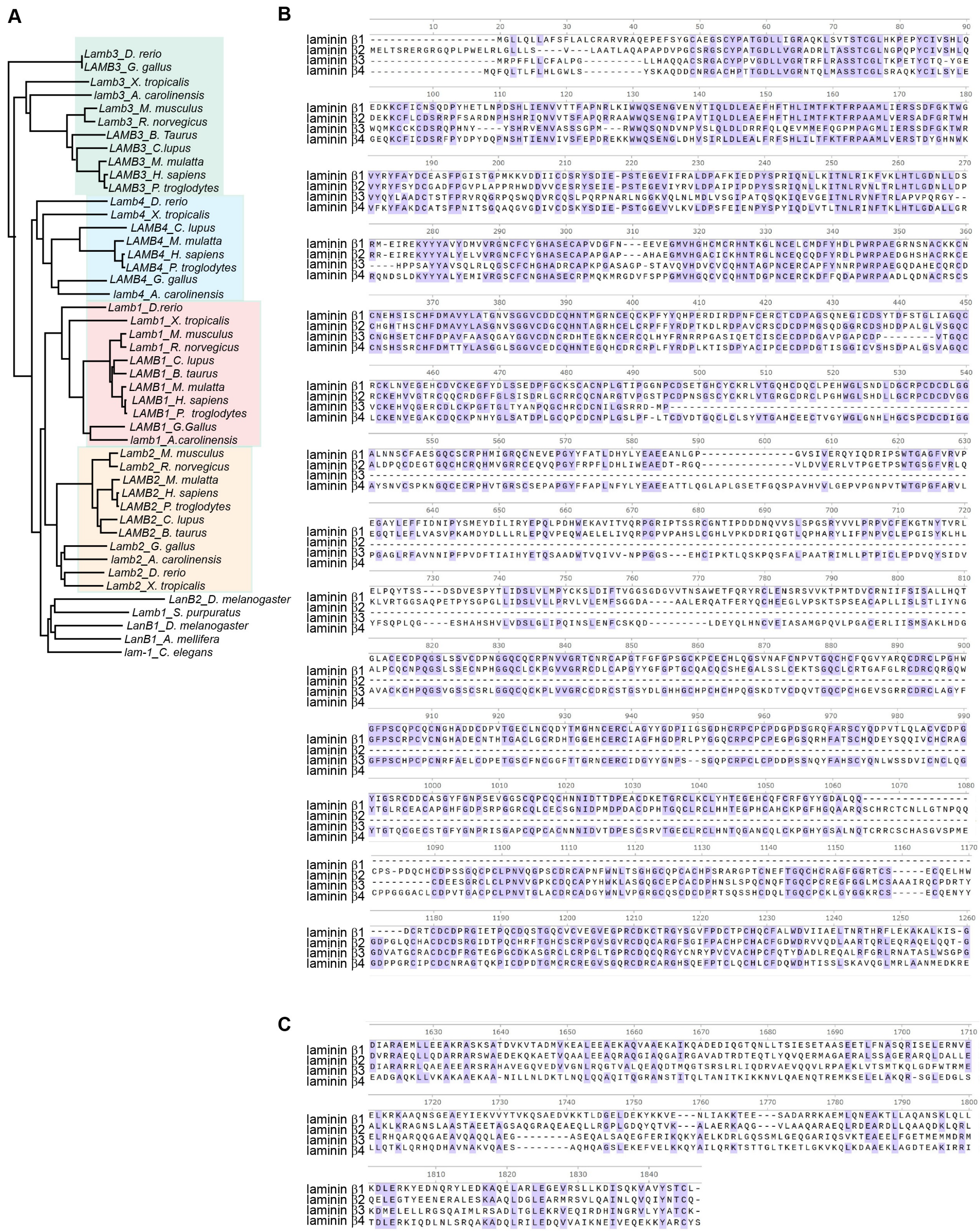

Fig. S1. Similarities between *LAMB* genes. A) Dendrogram of the *LAMB* genes among species. B and C) Alignment of regions in the B) N-terminal and C) C-terminal domains of the amino acid sequences of the human laminin β chains.

Development • Supplementary information

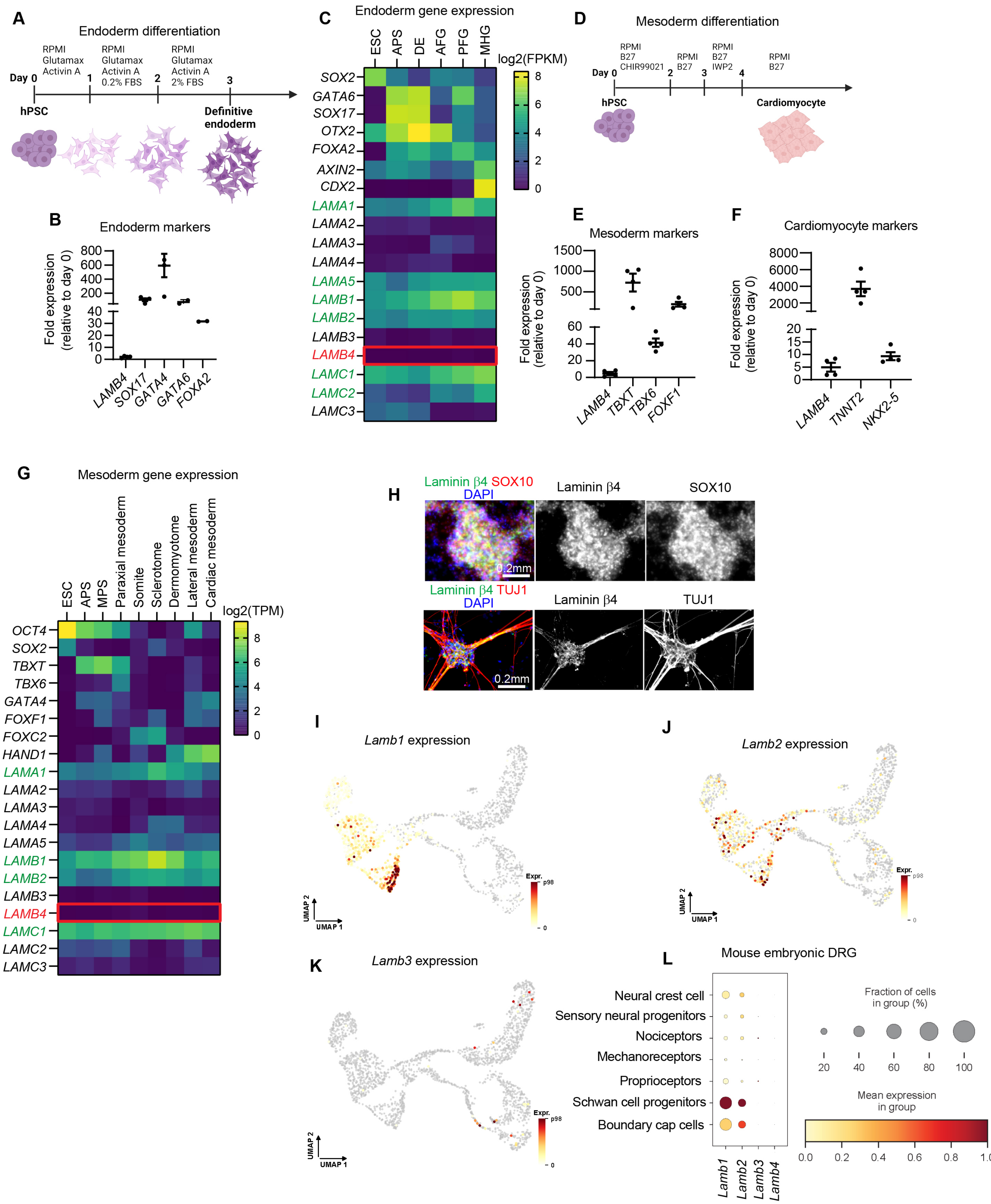

Development • Supplementary information

Fig. S2. *LAMB4* is not expressed in mesoderm and endoderm. A) Schematic of the endoderm differentiation protocol. B) Gene expression of endoderm markers. hPSC-ctr-H9 cells were differentiated into endoderm and RNA was isolated on day 3. mRNA levels were measured using RT-qPCR (n=2-4 biological replicates). C) *LAMB4* expression during endoderm differentiated from hPSCs. D) Schematics of the mesoderm differentiation protocol. E) Expression of early mesoderm marker. F) Expression of cardiomyocyte-related genes. Previously published RNAseq data was analyzed to assess the expression of laminin chains. G) *LAMB4* expression from mesoderm and cardiomyocytes differentiated from hPSCs. Highly expressed laminin genes are shown in green. *LAMB4* shown in red. H) Laminin β4 expression in NCCs (top row) and in SNs (bottom row). Day 12 NCCs differentiated from hPSC-ctr-H9 cells were fixed and stained for laminin β4, SOX10, and DAPI, for SNs hPSC-ctr-H9 SNs were fixed on day 20 and stained for the laminin β4, TUJ1, and DAPI. I-J) Uniform Manifold Approximation and projection (UMAP) embeddings of the embryonic murine DRG (E9.5-E12.5) displaying the spatial expression of Lamb1, Lamb2, and *Lamb3*. K) Comparative dot plot displaying the normalized expression and penetrance of *Lamb1*, *Lamb2, Lamb3*, and *Lamb4* across annotated neural and glial cell populations. Dot size represents the penetrance (percentage of cells expressing the transcript), and color intensity indicates the mean normalized expression level within expressing cells. Note the complete absence of *Lamb4* transcripts across all clusters.

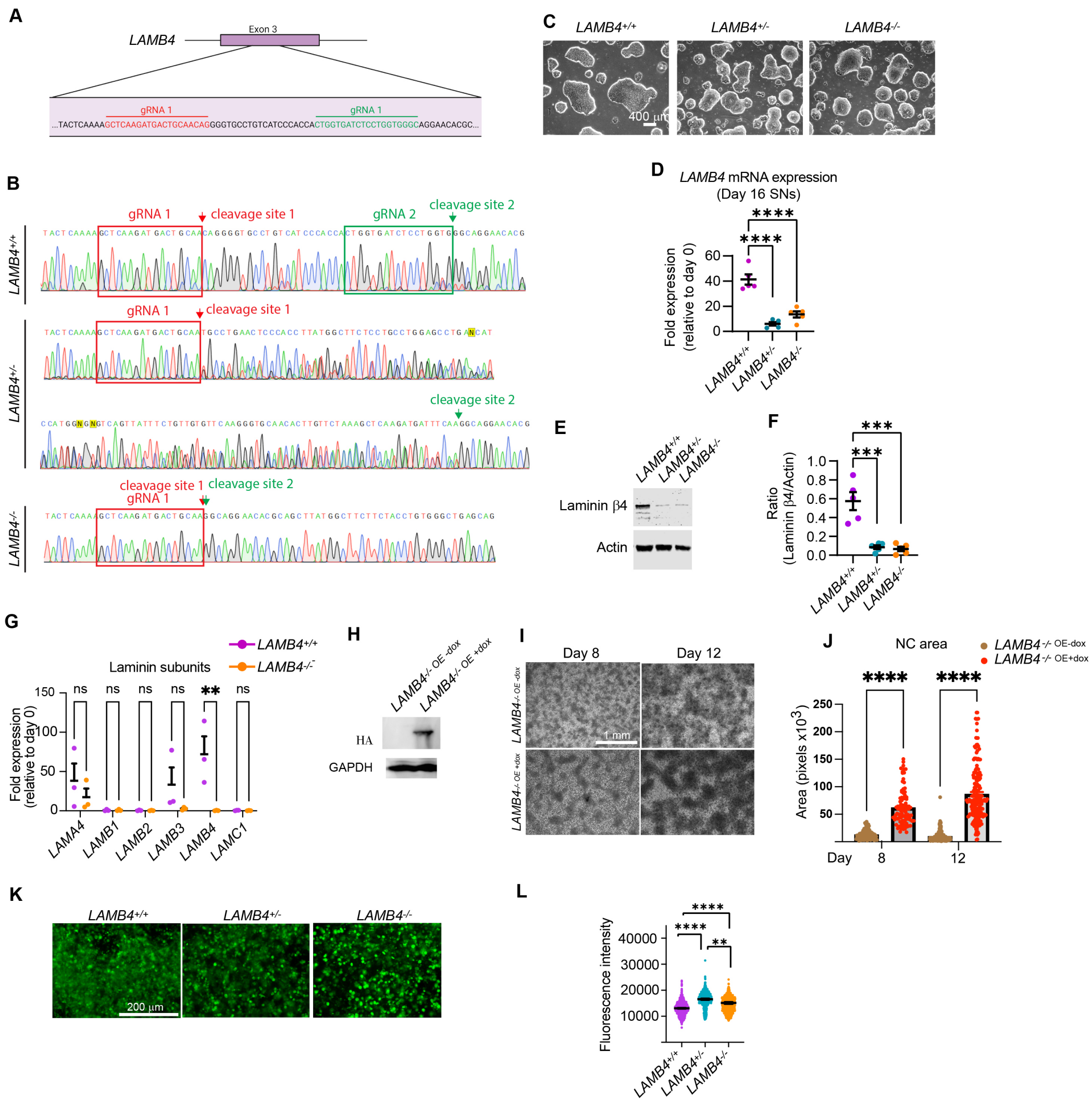

Fig. S3. LAMB4 editing strategy and characterization. A) Schematics of the strategy used to edit LAMB4 by CRISPR/Cas9. B) Sequencing results of *LAMB4+/+*, *LAMB4+/-*, and *LAMB4-/-* hPSCs. C) Characterization of *LAMB4+/+*, *LAMB4+/-*, and *LAMB4-/-* hPSCs by brightfield microscopy. D) LAMB4 expression in SNs differentiated from *LAMB4+/+*, *LAMB4 +/-*, and *LAMB4-/-* hPSCs. RNA was isolated on day 16 and LAMB4 expression was measured by RT-qPCR (n=5 biological replicates). E) Laminin β4 levels in *LAMB4+/+, LAMB4+/-*, and *LAMB4-/-* SNs. Total protein of day 20 SNs was immunoblotted for laminin β4 and actin. F) Measuring of signal intensity of the immunoblots from F) (n=5 biological replicates). G) Expression of laminin chains subunits. RNA of day 16 *LAMB4-/-* SNs was

Development • Supplementary information

isolated, and mRNA expression of laminin chains was assessed by RT-qPCR (n=3 biological replicates). H) Confirmation of *LAMB4* overexpression in *LAMB4-/-* OE cells. After 48 hours of doxycycline treatment in iPSCs, HA expression from *LAMB4-/-* OE -dox and *LAMB4-/-* OE +dox cells was assessed by immunoblotting to confirm doxycycline-induced *LAMB4* expression. I) Brightfield images of colonies of *LAMB4-/-* OE -dox and *LAMB4-/-* OE +dox in the absence and presence of 1 µg/ml doxycycline in media at NCCs stage (day 8 and day 12) showing the NCCs clusters. J) Quantification of the area of NCC-clusters in I). K) NCC cells of *LAMB4+/+, LAMB4+/-*, and *LAMB4-/-* at d12 stained with Caspase-3 L) Caspase-3 signal intensity in *LAMB4* mutant NCCs. Caspase-3 fluorescence intensity was measured from K). For D), F), G), and K), one-way ANOVA followed by Tukey's multiple comparisons test.Two-way ANOVA followed by Tukey's multiple comparisons test. For J) unpaired two-tailed t-test with Welch's correction. ns, non-significant; **P*<0.05, ***P*<0.01, ****P*<0.001, *****P*<0.0001. Graphs show mean ± SEM.

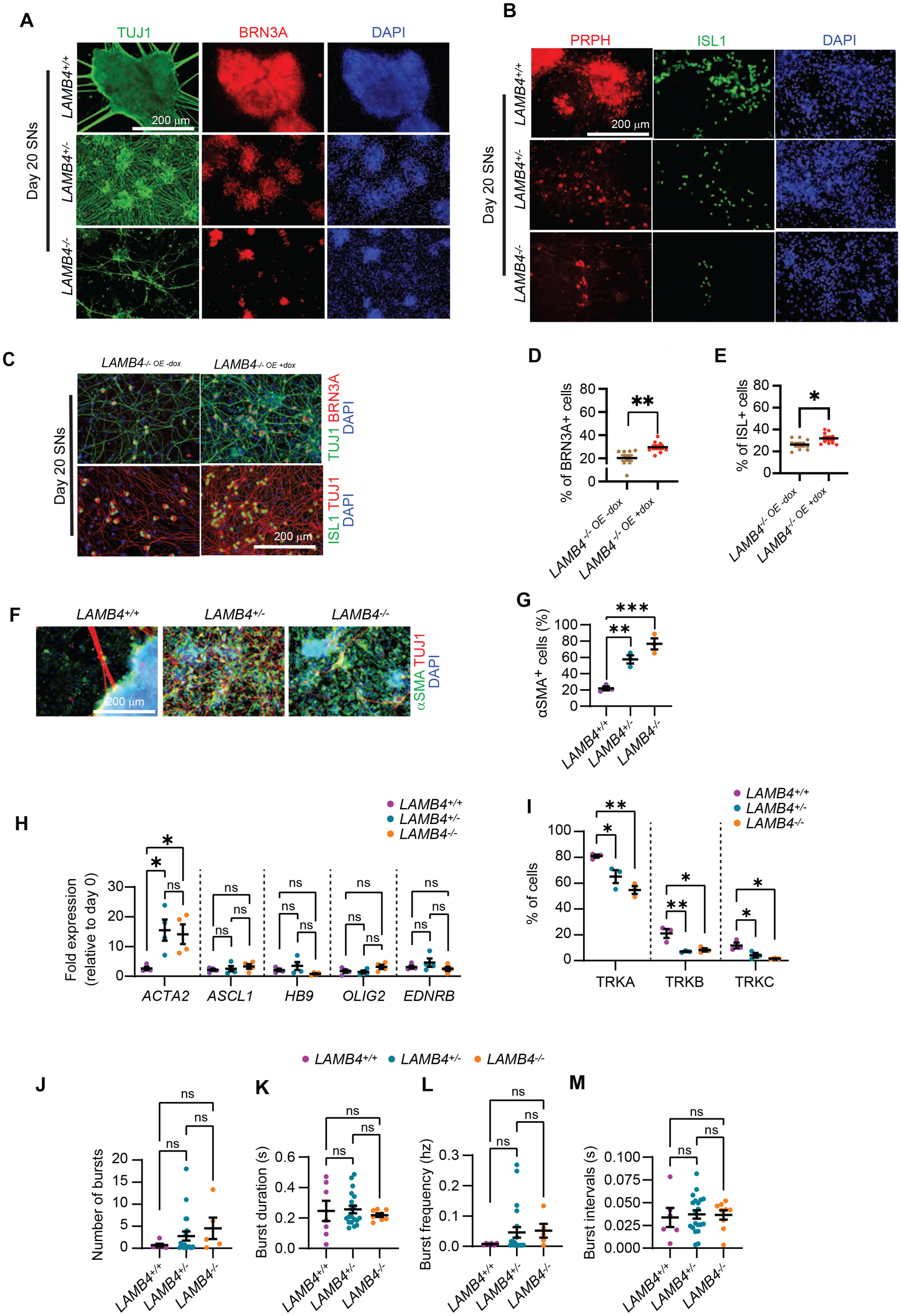

Development • Supplementary information

Fig. S4. Characterization of LAMB4 mutant SNs. A) and B) Expression of SN markers upon loss of *LAMB4*. These panels show individual fluorescence channels for the same merged images presented in Fig. 4B. *LAMB4+/+, LAMB4+/-*, and *LAMB4-/-* SNs were fixed on day 20 and stained for peripheral neuron markers (TUJ1 and PRPH) and SN markers (BRN3A, ISL1). Nuclei were stained with DAPI. C) Expression of SN markers in the absence and presence of 1 µg/ml doxycycline treatment in *LAMB4-/-* OE -dox and *LAMB4-/-* OE +dox. SNs were fixed on day 20 and stained for peripheral neuron marker (TUJ1) and SN markers (BRN3A, ISL1). Nuclei were stained with DAPI. D) Quantification percentage of BRN3A+ cells in C). Normalized to DAPI. E) Quantification percentage of ISL1+ cells in C). Normalized to DAPI. F) Expression of markers upon loss of *LAMB4*. *LAMB4*$^{+/+}$, *LAMB4*$^{+/-}$, and *LAMB4*$^{-/-}$ SNs were fixed on day 20 and stained for the non-neural ectoderm marker α-Smooth Muscle Actin (αSMA). Nuclei were stained with DAPI. G) Percentage of αSMA+ cells from F) over DAPI was plotted. H) Expression of genes not expressed in SNs. RNA of day 20 SNs differentiated from *LAMB4+/+*, *LAMB4+/-*, and *LAMB4-/-* hPSCs was isolated. Gene expression was measured by RT-qPCR (n=4 biological replicates). I) Percentage of cells expressing TRK proteins. Day 20 SNs were stained with TRKA, TRKB, and TRKC fluorescently-tagged antibodies and analyzed by flow cytometry (n=3 biological replicates). J-M) Measurement of electrical activity of *LAMB4+/+*, *LAMB4+/-*, and *LAMB4-/-* SNs. J) Number, K) duration, L) Frequency, and M) intervals of bursts were measured. Each dot represents the mean firing rate of 6 wells measured over 40 days (n=4 biological replicates). For D) and E) unpaired two-tailed t-test with Welch's correction. For H), one-way ANOVA followed by Dunnett's multiple comparisons test. For G), I), J), K), L), and M) one-way ANOVA followed by Tukey's multiple comparisons test. ns, non-significant; **P*<0.05, ***P*<0.01, ****P*<0.001, *****P*<0.0001. Graphs show mean ± SEM.

Development • Supplementary information

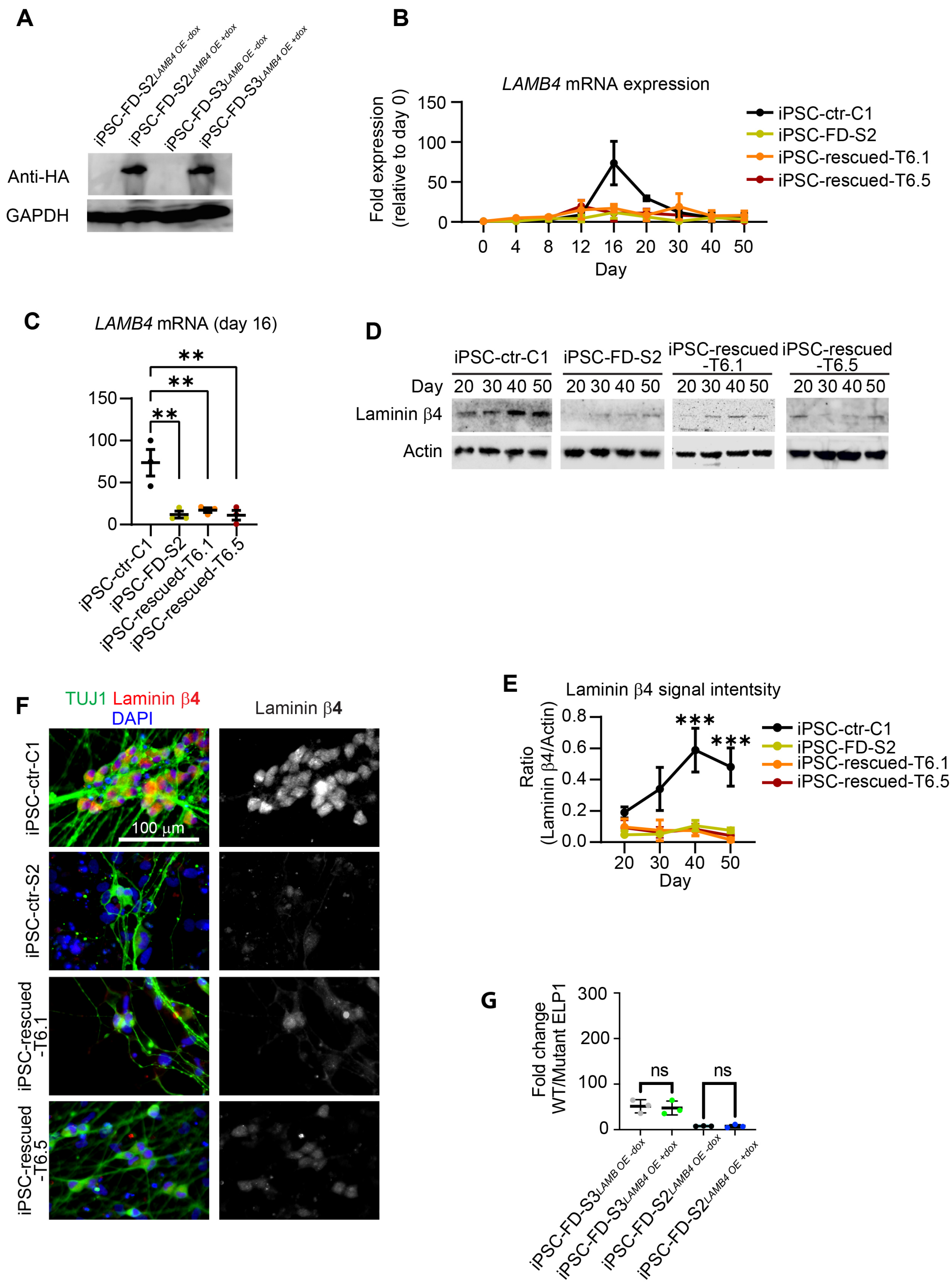

Development • Supplementary information

Fig. S5. LAMB4 expression is not dependent on ELP1. A) Confirmation of *LAMB4* overexpression in *LAMB4-/- OE* in severe FD iPSCs. After a 48-hour doxycycline treatment, iPSC-FD-S2 *LAMB4 OE dox* and iPSC-FD-S3 *LAMB4 OE dox* iPSCs were lysed and immunoblotted for HA tag to confirm doxycycline-induced *LAMB4* expression. B) LAMB4 expression in SNs differentiated from ELP1-rescued iPSCs of severe FD patients. *ELP1+/-* severe FD iPSCs (iPSCrescued-T6.1 and iPSC-rescued-T6.5), one severe FD iPSC line (S2), and one healthy iPSC control line (C1) were differentiated into SNs. Total RNA was isolated at the indicated times and gene expression was measured by RT-qPCR (n=3 biological replicates). C) *LAMB4* expression by SNs on day 16 shown in B) is shown (n=3 biological replicates). D) Laminin β4 expression during SN development. iPSC-rescued-T6.1, iPSC-rescued-T6.5, iPSC-FD-S2, and iPSC-ctr-C1 cells were differentiated into SNs. Lysates were collected on the indicated days and were immunoblotted for laminin β4 and actin. E) Quantification of signal intensity of immunoblots shown in D) (n=3 biological replicates). F) Laminin β4 in *ELP1*-rescued severe FD SNs. iPSC-rescuedT6.1, iPSC-rescued-T6.5, iPSC-FD-S2, and iPSC-ctr-C1 hPSCs were differentiated into SNs. Cells were fixed on day 20 and stained for laminin β4, TUJ1, and DAPI. G) RT-qPCR analysis for the ratio of WT to mutant ELP1 splicing in NCCs in iPSC-FD-S3*LAMB4 OE dox* and iPSC-FD-S2*LAMB4 OE dox* with and without doxycycline. For C) and G), one-way ANOVA followed by Tukey's multiple comparisons test. For F), two-way ANOVA followed by Šídák's multiple comparisons test. ns, non-significant; **P*<0.05, ***P*<0.01, ****P*<0.001, *****P*<0.0001. Graphs show mean ± SEM.

Development • Supplementary information

Table S1. List of cell lines we used in the study with their respective mutations

Name of the cell line	Disease phenotype in patient	*ELP1* Mutation	*LAMB4* mutation
hPSC-ctr-H9	healthy control	wild type	wild type
iPSC-ctr-C1	healthy control	wild type	wild type
iPSC-FD-M2	mild	homozygous point variation (Splicing donor intron 20) T>C	wild type
iPSC-FD-S1	severe	homozygous point variation (Splicing donor intron 20) T>C	Heterozygous missense variant (exon 19) G>T
iPSC-FD-S2	severe	homozygous point variation (Splicing donor intron 20) T>C	Heterozygous missense variant (exon 29, shared with S3) C>T
iPSC-FD-S3	severe	homozygous point variation (Splicing donor intron 20) T>C	Heterozygous missense variant (exon 29, shared with S2) C>T
iPSC-rescued-T6.1	healthy	genetically rescued heterozygous point variation(Splicing donor intron 20) T>C (parent line iPSC-FD-S2)	Heterozygous missense variant (exon 29, shared with S3) C>T
iPSC-rescued-T6.5	healthy	genetically rescued heterozygous point variation(Splicing donor intron 20) T>C (parent line iPSC-FD-S2)	Heterozygous missense variant (exon 29, shared with S3) C>T

Development • Supplementary information

Table S2. List of primers used in this study

Gene	Forward sequence	Reverse sequence
SOX10	CCAGGCCCACTACAAGAGC	CTCTGGCCTGAGGGGTGC
P75NTR	CCTCATCCCTGTCTATTGCTCC	GTTGGCTCCTTGCTTGTTCTGC
NGN1	GCCTCCGAAGACTTCACCTACC	GGAAAGTAACAGTGTCTACAAAGG
NGN2	CAAGCTCACCAAGATCGAGACC	AGCAACACTGCCTCGGAGAAGA
BRN3A	AGTACCCGTCGCTGCACTCCA	TTGCCCTGGGACACGGCGATG
RUNX1	CCACCTACCACAGAGCCATCAA	TTCACTGAGCCGCTCGGAAAAG
RUNX3	GGCAATGACGAGAACTACTCCG	GATGGTCAGGGTGAAACTCTTCC
TRKA	CACTAACAGCACATCTGGAGACC	ACAGTCAGCTCAAGCCAGACAC
TRKB	ACAGTCAGCTCAAGCCAGACAC	GTCCTGCTCAGGACAGAGGTTA
TRKC	CCGACACTGTGGTCATTGGCAT	CAGTTCTCGCTTCAGCACGATG
TBXT	CCTTCAGCAAAGTCAAGCTCACC	TGAACTGGGTCTCAGGGAAGCA
TBX6	TCATCTCCGTGACAGCCTACCA	CCGCAGTTTCCTCTTCACACGG
FOXF1	CAGCCTCACATCACGCAAGG	AGCCGAGCTGCAAGGCATC
TNNT2	AAGAGGCAGACTGAGCGGGAAA	AGATGCTCTGCCACAGCTCCTT
NKK2-5	AAGTGTGCGTCTGCCTTTCCCG	TTGTCCGCCTCTGTCTTCTCCA
GAPDH	GTCTCCTCTGACTTCAACAGCG	ACCACCCTGTTGCTGTAGCCAA
LAMA1	GAAGGTGACTGGCTCAGCAAGT	AGGCGTCACAACGGAAATCGTG
LAMA2	GGCAATCTGAATACACTCGTGAC	TGTGTTGGTCCTCTCAGCATCC
LAMA3	TAGAGGAAGCCTCTGACACAGG	CCGATAGTATCCAGGGCTACAAC
LAMA4	GAGATGACTCTCTGCTGGACCT	AGTTCCAGGCAGCCAACAAAGC
LAMA5	AACCAGATGAGCATCACATTCCTG	ACAGTGTTGCGCGTCTCCGTAT
LAMB1	GAGGTGTCTCAAGTGCCTGTAC	ACTGGCAGTCAGAGCCGTTACA
LAMB2	GCGGACTTGTTCTGAGTGCCAA	ACCTGTGAAGCGGTGACACTGA
LAMB3	GTCACAGAGCAGGAGGTGGCT	GCTTCTGTCAAGACTCTCCAGG
LAMB4	GTGGAGGCTTTACAACTGGCAG	GGATCATCTGGACACAGGCAAG
LAMC1	CTGTGAGGTCAACCACTTTGGG	AGCCTTCTCTGCATTCACAGCG
LAMC2	TACAGAGCTGGAAGGCAGGATG	GTTCTCTTGGCTCCTCACCTTG
LAMC3	CTGTAACCAGCATGGCACCTGT	ACCTGGCAAACAGCGTTCACAG
